# Human Hsp70 paralogs display selective J-domain interactions tuning proteostasis under stress

**DOI:** 10.1073/pnas.2534903123

**Published:** 2026-08-18

**Authors:** Roni Suhler, Lars J. W. van Beurden, Merav D. Shmueli, Ofrah Faust, Rina Rosenzweig

**Affiliations:** ^a^ Department of Chemical and Structural Biology, Weizmann Institute of Science, Rehovot 760001, Israel; ^b^ Department of Systems Immunology, Weizmann Institute of Science, Rehovot 760001, Israel

**Keywords:** molecular chaperones, protein homeostasis, protein folding and aggregation, NMR, Hsp70 and J-domain proteins

## Abstract

Hsp70 chaperones are central regulators of proteostasis, mediating folding, refolding, degradation, and aggregation-prevention. Their activity is tuned by the diverse J-domain proteins (JDPs), which both recruit client proteins and stimulate Hsp70 ATP hydrolysis via interaction with a conserved J-domain. The cytosol contains four Hsp70 paralogs—the stress-inducible HSPA1A/B and HSPA6, and the constitutively expressed HSPA8. Whether these act redundantly or carry distinct cellular functions has remained unresolved. Here, we systematically map the interactions of cytosolic Hsp70s with broad-specificity JDPs to elucidate how paralog identity shapes cellular function. We found that despite the high conservation of the JDP–Hsp70 interaction sites, the affinities of these interactions and their functionality varied greatly. HSPA8 behaves as a generalist, engaging all JDP classes with comparable affinity, consistent with its housekeeping role. By contrast, HSPA1 preferentially binds canonical Class A and B JDPs, while showing only weak binding to Class B′. Therefore, under stress, HSPA1 pairs only with Class A/B JDPs to support robust protein refolding, while freeing Class B′ to suppress protein aggregation in an Hsp70-independent manner. Most unexpectedly, HSPA6, the most stress-inducible paralog, binds selectivity to Class B JDPs, losing interactions with both Class A and B′. Thus, under severe stress, HSPA6 works exclusively with Class B JDPs to ensure ATP-dependent protein repair, while freeing Class A and B′ JDPs to act independently of Hsp70 to protect damaged/misfolded proteins. Our findings reveal an evolved hierarchy of paralog-specific JDP couplings that dynamically rewires the Hsp70 network from active repair to protection during stress.

## Significance

Proteostasis depends on the coordinated actions of Hsp70 chaperones and their diverse J-domain protein (JDP) cochaperones, yet whether these act redundantly or carry distinct cellular functions has remained unresolved. Here, we uncover that despite being highly conserved, the J-domain exhibits subtle sequence-level differences across different JDP classes, which tune the affinity and specificity of JDPs for Hsp70 paralogs. As cellular stress increases, stress-inducible Hsp70s interact with an increasingly selective subset of JDPs, effectively releasing other broad-specificity JDPs to function as stand-alone chaperones, independently of Hsp70, protecting cellular clients from misfolding and aggregation. Together, these results reveal an evolved hierarchy of paralog-specific JDP couplings that dynamically rewires the Hsp70 network during stress from adenosine triphosphate-dependent repair to protective containment, ensuring robust proteostasis.

**Fig. 1.**
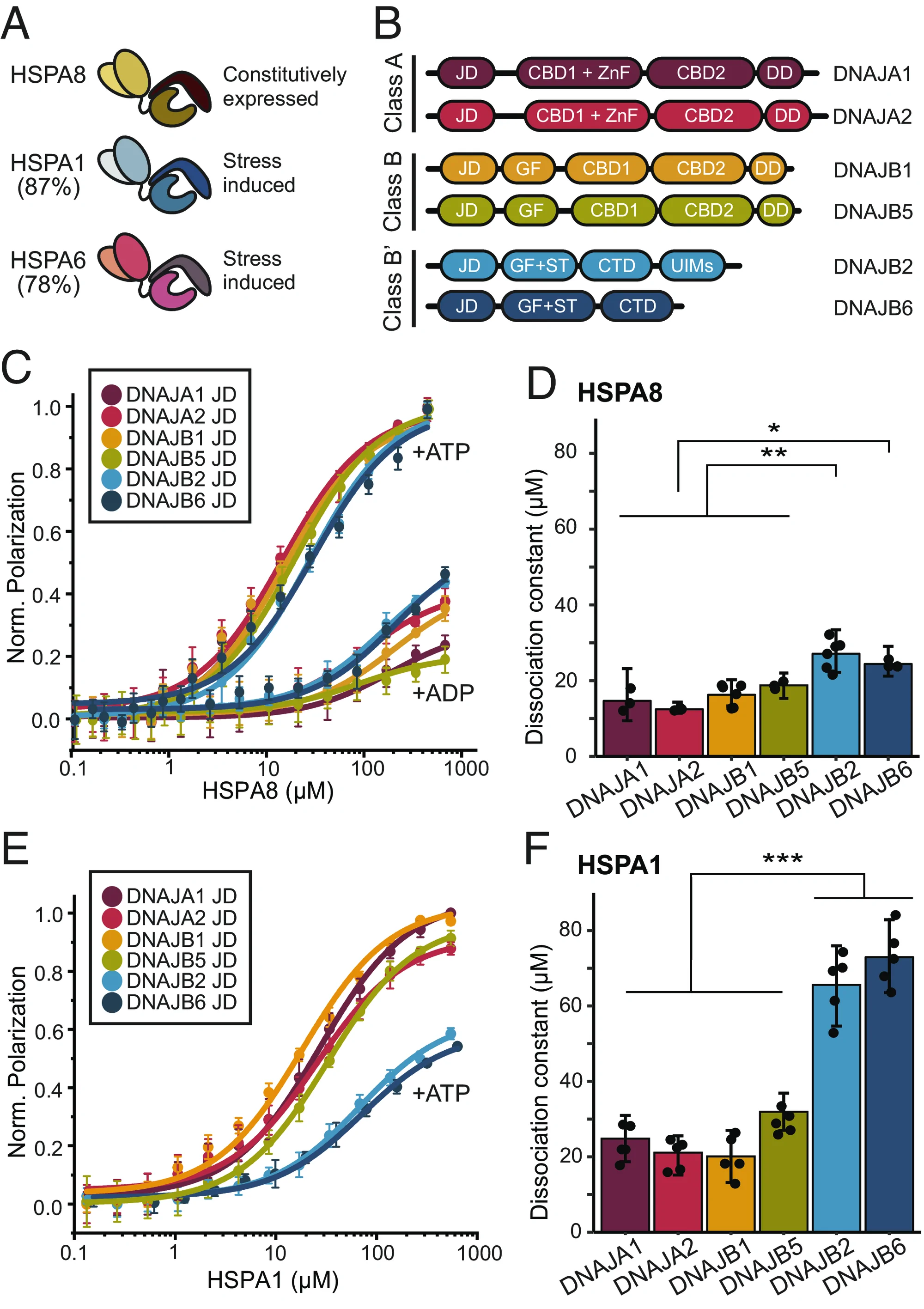
Interaction of cytosolic Hsp70s with broad-specificity JDP J-domains. (*A*) Schematic illustration of the cytosolic Hsp70 paralogs HSPA8, HSPA1, and HSPA6 chaperones and their expression patterns. Sequence identity to HSPA8 is indicated in brackets. (*B*) Domain organization of representative Class A (DNAJA1, DNAJA2), Class B (DNAJB1, DNAJB5), and Class B′ (DNAJB2, DNAJB6) JDPs used in this study. JD- J-domain; CBD—client binding domain; ZnF—zinc finger domain; DD—dimerization domain; GF—glycine/phenylalanine-rich region; ST—serine/threonine-rich region; CTD—C-terminal domain; UIMs—ubiquitin interaction motifs. (*C* and *E*) Fluorescence polarization measurements of DNAJA1 (maroon), DNAJA2 (red), DNAJB1 (yellow), DNAJB5 (olive), DNAJB2 (blue), and DNAJB6 (dark blue) J-domains binding to HSPA8 in the presence of ADP or ATP (*C*) or to ATP-HSPA1 (*E*). Data points represent the mean ± SD of three independent measurements. (*D* and *F*) Bar plots showing the dissociation constants (K_D_) for J-domains binding to HSPA8 (*D*) or HSPA1 (*F*) in the presence of ATP. Individual data points represent independent fluorescence polarization titrations for 3 to 5 independent measurements. Bars indicate the mean K_D_, and error bars represent the corresponding 95% CI calculated from log-transformed K_D_ values. Statistically significant changes are indicated with an asterisk (using one-way ANOVA on log-transformed K_D_ values followed by Tukey–Kramer post hoc analysis).

The 70-kDa heat shock proteins (Hsp70s) are central regulators of protein homeostasis, facilitating folding, refolding, disaggregation, and degradation of a wide variety of protein clients (1–4). In the human cytosol, the Hsp70 family includes four abundant paralogs: the constitutively expressed HSPA8 (Hsc70), and three stress-inducible paralogs, HSPA1A, HSPA1B, and HSPA6 (HSP70B′) (5–7) (Fig. 1*A*). HSPA1A and HSPA1B are nearly identical proteins expressed at low basal levels but strongly upregulated upon stress (8). In contrast, HSPA6 is a late-evolutionary paralog, virtually absent under basal conditions and robustly induced only during severe proteotoxic stress (6, 9–12). Despite their high sequence similarity, it remains unclear whether these Hsp70s are functionally redundant or have evolved distinct and specialized cellular roles in maintaining proteostasis.

The activity and client specificity of Hsp70s are largely determined by their interaction with J-domain proteins (JDPs, or Hsp40s), a diverse and functionally versatile family of chaperones that both recruit clients and stimulate the ATPase activity of Hsp70s to drive client engagement (4, 13–15). The interaction between Hsp70s and JDPs is mediated primarily through the conserved ~70-residue J-domain (JD) (16), which binds the nucleotide-binding domain (NBD) and interdomain linker of Hsp70, allosterically enhancing ATP hydrolysis and promoting conformational transitions critical for client capture (17).

Human cytosolic JDPs are typically classified into four major groups, based on their structural features: the conserved Class A (e.g., DNAJA1, DNAJA2, DNAJA4) and Class B (e.g., DNAJB1, DNAJB4, DNAJB5); the structurally divergent noncanonical Class B′ (e.g., DNAJB2, DNAJB6, DNAJB7, DNAJB8); and the highly versatile Class C JDPs (4, 13–15). While all classes share a conserved J-domain, they differ in their client binding regions and accessory domains that define substrate specificity and cellular function (4). Canonical Class A and B JDPs are broad-specificity chaperones that cooperate with Hsp70 in protein folding, refolding, degradation, and disaggregation (18–24), whereas noncanonical Class B′ JDPs possess potent Hsp70-independent antiaggregation activity (25–30).

Although the general principles of Hsp70–JDP cooperation are well established, the extent to which specific JDPs preferentially interact with individual Hsp70 paralogs, their hierarchy of interactions, and how such selectivity shapes chaperone activity under stress remain largely unresolved. A few studies have suggested a functional divergence among cytosolic Hsp70 paralogs. HSPA1A and HSPA8 have partially overlapping but distinct clientomes and ATPase regulation (31–33), whereas HSPA6 has been reported to bind certain clients, such as unfolded p53, more efficiently than HSPA1A (6). Moreover, siRNA-mediated depletion studies suggest nonredundant roles for HSPA6 in supporting cell survival under extreme stress, compared with HSPA1 (6). Yet, these studies do not directly address the contribution of JDPs to Hsp70 functional specialization.

Here, we set out to systematically define the binding preferences of human cytosolic Hsp70 paralogs for a representative panel of broad-specificity JDPs from each major class (Fig. 1*B*). Using NMR spectroscopy, fluorescence polarization, and biochemical assays, we reveal that each Hsp70 paralog exhibits distinct JDP selectivity. HSPA8 shows broad affinity for all six selected JDPs, consistent with its constitutive generalist role. By contrast, the stress-inducible HSPA1 preferentially interacts with canonical Class A and B JDPs, while showing weak binding to noncanonical Class B′ proteins. Notably, as we find that Class B′ JDPs do not promote Hsp70-dependent protein refolding, their low affinity for HSPA1 ensures that under cellular stress conditions, they remain available to suppress aggregation in an Hsp70-independent manner. More strikingly, we find that HSPA6 binds exclusively to Class B JDPs, showing negligible interaction with Class A or Class B′ JDPs, despite the high conservation of their J-domain architecture. As HSPA6 expression is induced only under severe stress, this selective binding ensures that exclusively Class B JDPs engage with this Hsp70, while the highly potent aggregation suppressors of Class A and B′ act independently of Hsp70 to prevent client misfolding and aggregation, ensuring both immediate protection and efficient proteome repair.

Together, our findings demonstrate that, despite the J-domain–Hsp70 interface being one of the most conserved elements of the chaperone machinery and long assumed to be structurally and functionally invariant (34), subtle sequence and conformational variations within J-domains fine-tune their affinity for different Hsp70 paralogs. These subtle adaptations give rise to a functional hierarchy in which distinct Hsp70 paralogs selectively interact with different subsets of the JDP chaperones to balance Hsp70-dependent and Hsp70-independent activities, reflecting an evolved hierarchy within the stress-inducible chaperones.

## Results

### Interaction of HSPA8 (Hsc70) with the Broad Specificity JDP J-Domains.

The interaction between Hsp70s and JDPs is essential for Hsp70 function, as it stimulates Hsp70 ATP hydrolysis rates and facilitates client delivery. This crucial interaction is primarily mediated through the J-domain, triggering the large conformational change that activates the chaperone cycle. To systematically compare JDP binding preferences across cytosolic Hsp70 paralogs, we focused on the isolated J-domains, thereby eliminating contributions from client-binding domains and secondary interdomain interactions (4, 23, 35, 36).

We selected six broad specificity JDPs from three different classes: DNAJA1 and DNAJA2 (Class A), DNAJB1 and DNAJB5 (Class B), and DNAJB2 and DNAJB6 (Class B′, noncanonical Class B) (Fig. 1*B*). Among these JDPs, some have been shown to be stress-induced (6, 37)(38), while others are not. The J-domains from these JDPs are highly conserved in sequence and structure, sharing over 50% sequence identity and more than 75% similarity. All six J-domains and three human cytosolic Hsp70 paralogs (HSPA1, HSPA6, and HSPA8) were successfully expressed and purified, yielding highly pure proteins with functional J-domains (*SI Appendix*, Fig. S1 *A*–*C*, and Fig. S8 *E*).

To characterize the specificity and hierarchy of cytosolic Hsp70 and JDP protein–protein interactions, we first set out to quantitatively determine the affinity of each of the JDP J-domains for HSPA8 using fluorescence polarization. To this end, the J-domains were site-specifically labeled with Alexa-488 fluorophore, through a cysteine introduced at their C-terminus (*Materials and Methods*). Each labeled J-domain was titrated with increasing concentrations of ATPase-deficient HSPA8 T204A variant (Fig. 1*C*).

As expected, HSPA8–J-domain binding was strongly nucleotide-dependent. In the presence of ADP, HSPA8 exhibited only weak interactions with all six J-domains (Fig. 1*C*). In contrast ATP-bound HSPA8 T204A bound to all six JDPs with dissociation constants ranging from 12 to 27 µM. These values are in good agreement with the affinities reported for the bacterial DnaK-DnaJ system (39, 40). Notably, the canonical JDPs bound HSPA8 with higher affinity: DNAJA1 (K_D_ = 14.5 µM), DNAJA2 (K_D_ = 12.5 µM), DNAJB1 (K_D_ = 15.8 µM), and DNAJB5 (K_D_ = 18.8 µM), while the noncanonical Class B′ JDPs DNAJB2 and DNAJB6 showed moderately weaker binding, with dissociation constants of 27.1 µM and 24.4 µM, respectively (Fig. 1 *C* and *D* and *SI Appendix*, Table S1).

Considering the estimated intracellular concentrations of these JDPs under non-stress conditions (41, 42) and the affinity measurements, in the cell HSPA8 may preferentially engage the Class A JDP DNAJA1, followed closely by DNAJA2, DNAJB1, and DNAJB6 (*SI Appendix*, Table S3). Despite slightly stronger binding to the canonical JDPs, HSPA8 appears capable of interacting with all three JDP classes, suggesting a broad capacity to support various functions including de novo folding, refolding, complex remodeling, aggregation prevention, disaggregation, and degradation, all critical for maintaining cellular proteostasis.

### The Affinity between the J-Domains to HSPA1 Dictates Their Refolding Activity.

Next, we aimed to determine whether the stress-inducible HSPA1 displays a similar preference hierarchy for the JDPs as the constitutively expressed HSPA8. Since HSPA1A and HSPA1B isoforms are completely identical at the amino acid level (38), it allowed us to consider them as a single protein in functional analyses. We measured the binding affinities of HSPA1 T204A for the six JDP J-domains. Overall, HSPA1 displayed slightly weaker affinities for the canonical JDPs compared to HSPA8: DNAJA1 (25 µM), DNAJA2 (20 µM), DNAJB1 (20 µM), and DNAJB5 (32 µM) (Fig. 1 *E* and *F* and *SI Appendix*, Table S1). However, the most striking difference was observed with the noncanonical Class B′ JDPs, DNAJB2 and DNAJB6, which bound HSPA1 with significantly lower affinities: 66 µM and 73 µM, respectively (Fig. 1 *E* and *F* and *SI Appendix*, Table S1). These results suggest that, unlike HSPA8, HSPA1 likely does not engage Class B′ JDPs under physiological conditions, and primarily favors canonical JDPs (*SI Appendix*, Table S3).

**Fig. 2.**
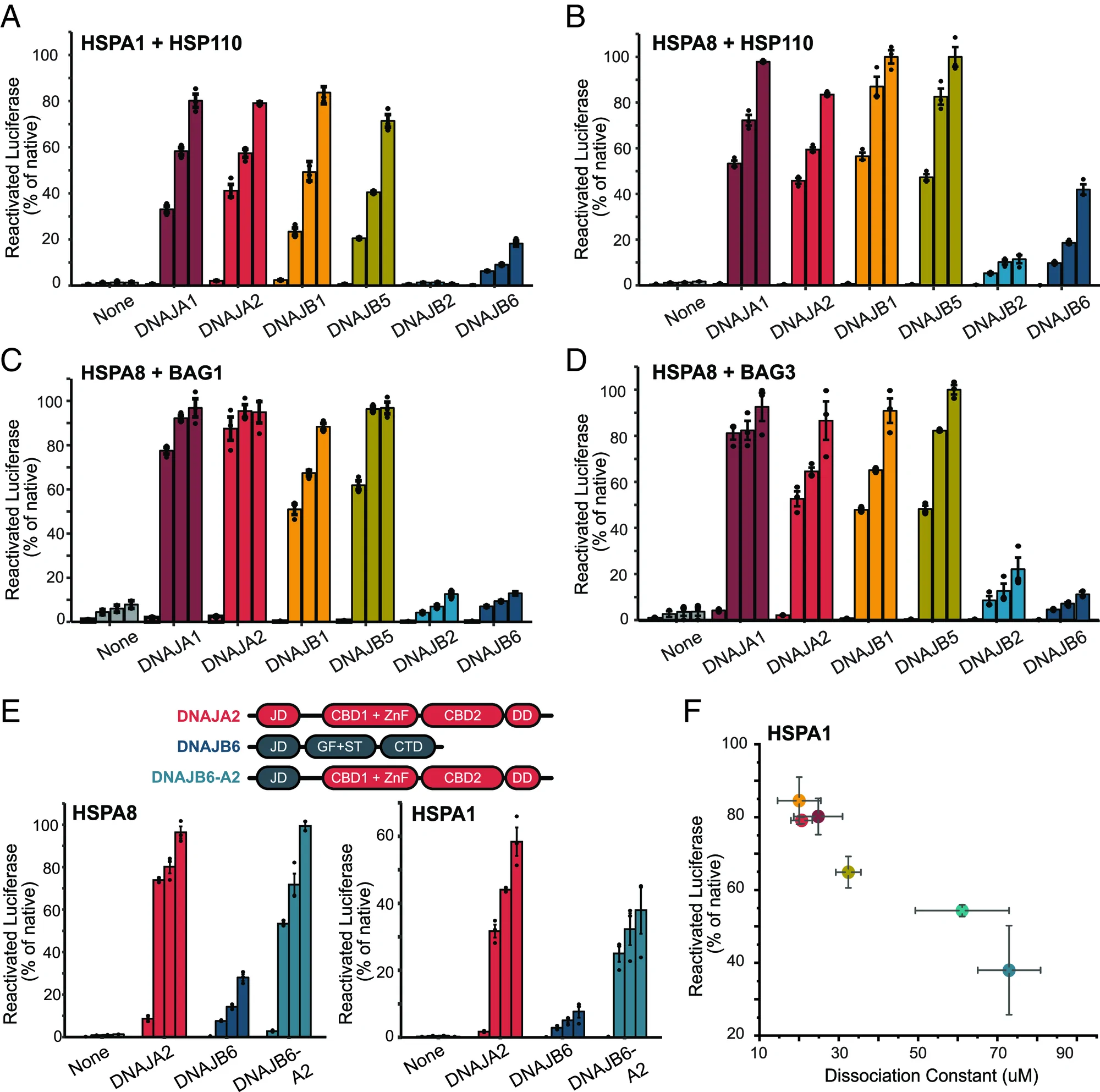
J-domain affinity for HSPA1 dictates chaperone refolding activity. (*A* and *B*) Refolding activity of FFL mediated by HSPA1 (*A*) or HSPA8 (*B*) together with HSP110, and in the absence (gray) or presence of DNAJA1 (maroon), DNAJA2 (red), DNAJB1 (yellow), DNAJB5 (olive), DNAJB2 (blue), or DNAJB6 (dark blue). FFL activity was measured at 0 min, 30 min, 60 min, and 120 min. Data points represent the mean ± SD of three independent measurements. (*C* and *D*) Refolding activity of FFL mediated by HSPA8 together with BAG1 (*C*) or BAG3 (*D*), and in the absence (gray) or presence of the indicated JDPs. (*E*) Domain organization of the wild-type (WT) DNAJA2 (red), WT DNAJB6 (dark blue), and the chimeric DNAJB6-A2 (dark turquoise), in which the DNAJB6 J-domain was fused to the client-binding domain of DNAJA2. FFL refolding by HSPA1 or HSPA8 in the presence of the indicated JDP variants. (*F*) Correlation between the dissociation constants (K_D_) of J-domain binding to HSPA1 and the ability of the corresponding full-length JDPs DNAJA1 (maroon), DNAJA2 (red), DNAJB1 (yellow), DNAJB5 (olive), DNAJB2-A2 (turquoise), and DNAJB6-A2 (dark turquoise) to support HSPA1-dependent refolding of FFL.

Intrigued by the differences in binding affinities, we wished to explore their functional implications. To this end, we measured the ability of HSPA1 to refold chemically denatured firefly luciferase (FFL) in the presence of each full-length JDP and a nucleotide exchange factor [NEF, Hsp110 (43)]. Canonical Class A and B JDPs (DNAJA1, DNAJA2, DNAJB1, and DNAJB5) supported efficient refolding by HSPA1, with FFL reactivation yields exceeding 80% within 120 min (Fig. 2*A*). In contrast, the noncanonical JDPs DNAJB2 and DNAJB6 showed only minimal refolding activity under identical conditions (Fig. 2*A*).

This lack of activity of the Class B′ JDPs could stem from their weaker J-domain–HSPA1 interactions, or alternatively, from inherent differences in the structure and function of their client-binding domains. The C-terminal domains (CTDs) of DNAJB2 and DNAJB6, which are involved in client binding, differ significantly from those of canonical JDPs (4, 44–46) (Fig. 1*B*) and may be structurally incompatible with misfolded luciferase, failing to promote its transfer to HSPA1 for refolding.

To distinguish between these possibilities, we repeated the FFL refolding assay with HSPA8, which binds all six JDP J-domains with relatively similar affinities (Fig. 1 *C* and *D*). Interestingly, even in the presence of HSPA8, DNAJB2, and DNAJB6 supported only limited refolding activity (Fig. 2*B*). To verify that this lack of refolding activity is not due to the nature of the nucleotide exchange factor (47–49), we repeated the refolding experiments using BAG1 and BAG3 as NEFs (Fig. 2 *C* and *D*). In both cases, only minimal refolding activity was measured for HSPA8 with both DNAJB2 and DNAJB6, while class A and B JDPs supported efficient refolding. Thus, the inability of the Class B′ JDPs to support Hsp70-dependent refolding is independent of the NEF used (Fig. 2 *C* and *D* and *SI Appendix*, Fig. S1 *D* and *E*).

Last, to ensure that this reduced activity was not client-specific, we performed parallel refolding assays with chemically denatured citrate synthase in the presence of each full-length JDP and a nucleotide exchange factor (NEF, Hsp110). Consistent with the FFL results, efficient refolding was detected only with the canonical class A and B JDPs and not with the noncanonical class B′ chaperones (*SI Appendix*, Fig. S1*F*). Thus, the reduced functional capacity of DNAJB2 and DNAJB6 is most likely due to the nature of their client-binding domains, which are incompatible with misfolded client recognition and therefore do not support refolding.

To directly test this hypothesis, we designed two chimeric JDP constructs, DNAJB6-A2 and DNAJB2-A2, in which the J-domains of DNAJB2 or DNAJB6 were fused to the client-binding domain of DNAJA2 (Fig. 2*E*). These chimeras were tested in the luciferase refolding assay alongside their WT counterparts and DNAJA2 (Fig. 2*E* and *SI Appendix*, Fig. S1*G*). Remarkably, both chimeras fully restored the refolding activity of HSPA8 to levels comparable to the WT DNAJA2 (Fig. 2 *E*, *Left* and *SI Appendix*, Fig. S1 *G*, *Left*), confirming that the inability of Class B′ JDPs to support refolding arises from the nature of their client-binding domains, rather than from reduced J-domain–Hsp70 interaction. In contrast, neither chimera restored full HSPA1 FFL refolding activity, and their refolding efficiencies closely correlated with the binding affinities of the J-domains for the HSPA1 chaperone (Fig. 2*F*), revealing a direct relationship between the strength of the J-domain–Hsp70 interaction and the overall refolding capacity of the chaperone system.

### A Single Residue Is Sufficient to Tune the Interaction between the J-Domains and HSPA1.

Given that HSPA1 displays a clear preference for canonical Class A and B JDPs over noncanonical Class B′ JDPs and that these differences in affinity directly impact refolding efficiency, we sought to identify the structural basis underlying this specificity.

To this end, we first mapped the interaction interfaces between each J-domain and HSPA1 using NMR spectroscopy. All six J-domains were expressed with uniform ^15^N-labeling and analyzed by 2D ^1^H-^15^N TROSY-HSQC. The resulting spectra showed well-dispersed peaks, consistent with folded proteins. As no chemical shift assignments were previously available for the DNAJB5 J-domain, we carried out full backbone resonance assignment, successfully providing unambiguous assignment for 97% of nonproline residues (*SI Appendix*, Fig. S2*A*). Secondary structure propensities and structural modeling using TALOS and CS-Rosetta confirmed that DNAJB5 adopts a canonical J-domain fold (*SI Appendix*, Fig. S2 *B* and *C*).

To map the binding surfaces, we recorded ^1^H-^15^N TROSY-HSQC spectra for each J-domain in the absence or presence of unlabeled HSPA1 T204A (*SI Appendix*, Fig. S3). Upon addition of HSPA1, several residues in each J-domain exhibited peak broadening, indicative of binding. Mapping the intensity differences between the bound (I) and free (I_0_) states onto the J-domain structures revealed that, in all cases, the affected residues clustered at the end of the second α-helix, within the flexible loop containing the conserved HPD motif, and at the beginning of the third α-helix. Similar residues showed binding for all six J-domains, and the interaction interfaces were highly similar to those detected upon HSPA8 binding (*SI Appendix*, Fig. S4), highlighting the conserved nature of the J-domain–Hsp70 interactions.

**Fig. 3.**
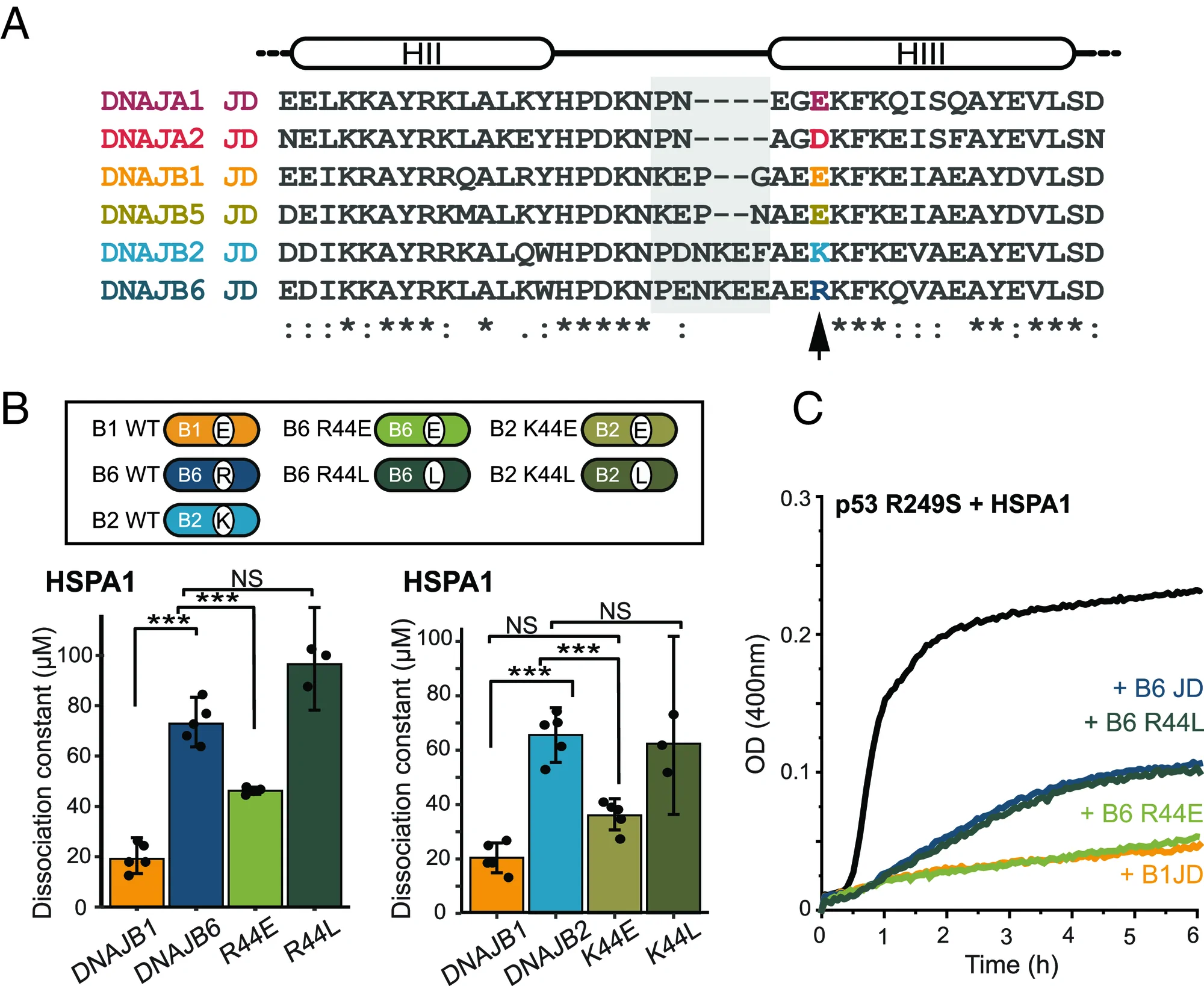
A single residue tunes the interaction between J-domains and HSPA1. (*A*) Clustal Omega sequence alignment of representative J-domains, highlighting residues identified by NMR as part of the HSPA1-binding surface. The key position that explains affinity differences toward HSPA1 is shown in bold and color. The positions of helices II and III are indicated above the alignment. (*B*) Bar plots showing the dissociation constants (K_D_) for HSPA1 binding to DNAJB1 JD (yellow), DNAJB6 JD (dark blue), DNAJB6 R44E (light green), DNAJB6 R44L (green) (*Left*), and to DNAJB1 JD (yellow), DNAJB2 JD (blue), DNAJB2 K44E (khaki), and DNAJB2 K44L (dark olive) (*Right*). Individual data points represent independent fluorescence polarization titrations for 3 to 5 independent measurements. Bars indicate the mean K_D_, and error bars represent the corresponding 95% CI calculated from log-transformed K_D_ values. Statistical significance was assessed using one-way ANOVA on log-transformed K_D_ values, followed by Tukey–Kramer post hoc analysis. (*C*) Aggregation prevention of mutants p53 R249S by HSPA1 alone (black) or in the presence of DNAJB1 JD (yellow), DNAJB6 JD (dark blue), DNAJB6 R44E JD (light green), and DNAJB6 R44L JD (green), monitored by light scattering.

Despite this conserved interaction surface, we hypothesized that subtle sequence differences could account for the observed variation in affinity toward HSPA1. We therefore performed a sequence alignment of the residues identified by NMR as part of the HSPA1-binding surface (Fig. 3*A*). This alignment revealed a high degree of conservation, particularly at residues we found to be critical for Hsp70 interaction. However, these sequences were not identical, and one residue located at the start of helix III differed consistently between canonical and noncanonical JDPs. Canonical Class A and B JDPs contain a negatively charged residue at this position (E43 in DNAJA1, DNAJB1, DNAJB4, and DNAJB5; D45 in DNAJA2), whereas noncanonical Class B′ JDPs contain a positively charged residue (K44 in DNAJB2 and R44 in DNAJB6) (Fig. 3*A*). As electrostatic interactions are known to influence J-domain–Hsp70 binding efficiency (34), we hypothesized that this single charge inversion may explain the weaker binding of Class B′ JDPs to HSPA1. Supporting this, an AlphaFold model of the HSPA1-DNAJB1 J-domain complex showed a potential salt bridge formation between E43 of the J-domain and K423 of HSPA1, which is lacking in the HSPA1–DNAJB6 J-domain complex (*SI Appendix*, Fig. S5 *A* and *C*). Moreover, comparing NMR spectra of DNAJB1 and DNAJB6 bound to HSPA1 revealed distinct interaction profiles at this position, suggesting that this residue engages HSPA1 in the canonical, but not in the noncanonical Class B′ JDPs (*SI Appendix*, Fig. S5 *B* and *D*).

To test whether this charge difference governs the affinity of HSPA1 to the J-domains, we generated J-domain point mutants in which the positive residue in DNAJB2 and DNAJB6 was replaced with a glutamate (K44E and R44E, respectively), mimicking the canonical J-domain sequences. Following purification and labeling of the proteins with Alexa-488 fluorophore, we measured binding affinities using fluorescence polarization, comparing the mutants to their WT counterparts and to the high-binding affinity DNAJB1 J-domain. Strikingly, both DNAJB2 and DNAJB6 mutants displayed a statistically significant increase in affinity for HSPA1 compared to their respective WT: DNAJB2 K44E improved from 66 to 36 µM, and DNAJB6 R44E improved from 73 to 46 µM, approaching the affinity of DNAJB1 (20 µM) (Fig. 3*B* and *SI Appendix,* Tables S1 and S2). In contrast, control mutations to leucine (K44L and R44L) showed no improvement in binding (Fig. 3*B* and *SI Appendix,* Tables S1 and S2), confirming the specific contribution of the electrostatic interaction.

AlphaFold structures of HSPA1–J-domain complexes show that residue E43 of DNAJB1 forms a salt bridge with K423 of HSPA1 (*SI Appendix*, Fig. S5*A*). To verify that the lack of this salt bridge is behind the lower affinity of the noncanonical class B′ JDPs for HSPA1, we have generated a K423E HSPA1 variant and measured its affinity for the J-domains using fluorescence anisotropy. Excitingly, this HSPA1 variant displayed a significantly lower affinity for the J-domains of the noncanonical class B′ JDPs than for class A and B (*SI Appendix*, Fig. S5*E* and Table S2), showing that the salt bridge formation indeed increases the affinity and governs HSPA1 specificity for the J-domains.

Together, these results demonstrate that a single charge reversal is sufficient to tune HSPA1 affinity and provide a molecular explanation for the observed interaction hierarchy.

### Affinity-Modulating Mutations Enhance Cochaperone Activity.

To determine whether these affinity-enhancing mutations influence JDP cochaperone activity, we assessed their effects on Hsp70-aggregation prevention. While Hsp70s can prevent protein aggregation independently, their activity is significantly enhanced by J-domains, which stimulate ATP hydrolysis and facilitate substrate engagement (50–53). We therefore tested the ability of HSPA1, in combination with WT and mutant J-domains, to prevent the aggregation of a destabilized p53 R249S mutant, a known Hsp70 client (6, 54) (*SI Appendix*, Fig. S5*F*). Importantly, since full-length JDPs also possess client-binding domains that can independently suppress aggregation, we used isolated J-domains to focus specifically on their interaction with HSPA1. We compared the aggregation prevention activity of the DNAJB6 WT JD, R44E mutant, and R44L control, alongside the DNAJB1 J-domain as a positive control. The high-affinity DNAJB6 R44E variant significantly enhanced the ability of HSPA1 to suppress p53 aggregation, reaching a level comparable to DNAJB1, whereas the WT and R44L mutant had a much milder effect (Fig. 3*C* and *SI Appendix*, Fig. S5*F*). These findings indicate that the affinity of J-domains for HSPA1 not only determines binding but also functionally modulates Hsp70 activity.

Overall, our results reveal a hierarchy of interaction between HSPA1 and the J-domains, and specifically lower affinity toward the noncanonical Class B′ J-domains. This affinity difference is tuned by a single residue exchange from a negative to a positive amino acid, which correlates with aggregation prevention and refolding activity of HSPA1. These results indicate that under stress conditions, when HSPA1 levels rise, it preferentially interacts with the canonical Class A and B JDPs, to facilitate refolding, aggregation prevention, and disaggregation functions. In parallel, the noncanonical Class B′ JDPs, which do not participate in Hsp70-dependent protein refolding, can prevent aggregation independently of Hsp70.

### The Stress-Induced HSPA6 Shows High Selectivity Only for the Canonical Class B JDPs.

**Fig. 4.**
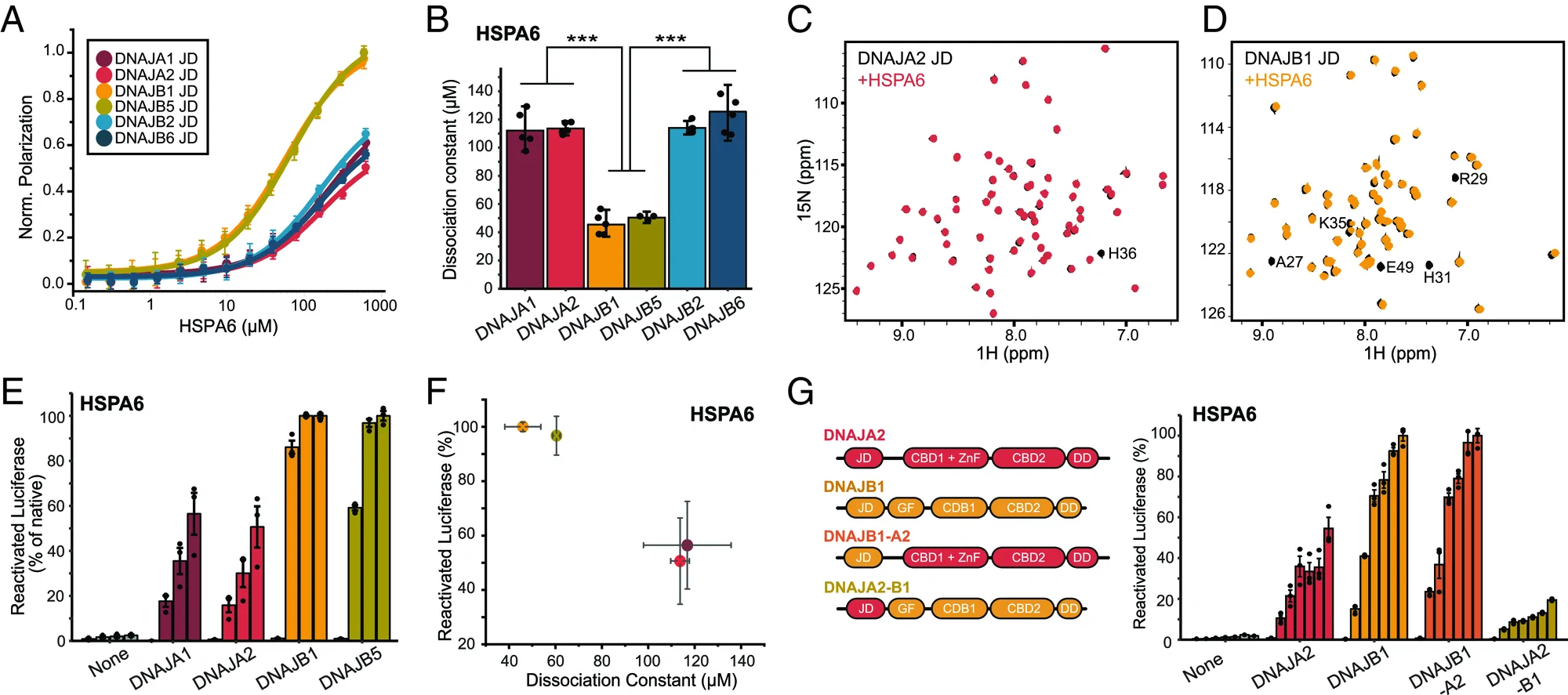
HSPA6 shows high selectivity for the canonical Class B JDPs. (*A*) Fluorescence polarization measurements of DNAJA1 (maroon), DNAJA2 (red), DNAJB1 (yellow), DNAJB5 (olive), DNAJB2 (blue), and DNAJB6 (dark blue) J-domains binding to HSPA6. (*B*) Bar plots showing the dissociation constants (K_D_) for the interactions between the indicated J-domains and ATP-bound HSPA6. Individual data points represent independent fluorescence polarization titrations for 3 to 5 independent measurements. Bars indicate the mean K_D_, and error bars represent the corresponding 95% CI calculated from log-transformed K_D_ values. Statistically significant differences are indicated with an asterisk (one-way ANOVA with Tukey–Kramer post hoc test). (*C* and *D*) ^1^H-^15^N HSQC spectra of 0.2 mM DNAJA2 (*C*) and DNAJB1 (*D*) J-domains alone (black) and in the presence of 0.05 mM HSPA6 T205A (red and yellow, respectively). Addtion of HSPA6 induces substantial peak broadening only to DNAJB1 JD and not to DNAJA2 JD. (*E*) Refolding activity of FFL mediated by HSPA6 and HSP110, in the absence (gray) or presence of DNAJA1 (maroon), DNAJA2 (red), DNAJB1 (yellow), and DNAJB5 (olive). (*F*) Correlation between the dissociation constants (K_D_) of the J-domains binding to HSPA6, and the ability of the corresponding full-length JDPs to support HSPA6-dependent FFL refolding activity. (*G*) Domain organization of WT DNAJA2 (red), WT DNAJB1 (yellow), DNAJB1-A2 chimera (orange) in which DNAJB1 J-domain was fused to the client-binding domain of DNAJA2, and DNAJA2-B1 chimera (dark gold) in which DNAJA2 J-domain was fused to the client-binding domains of DNAJB1. FFL refolding by HSPA6 and Hsp110 in the presence of the indicated JDPs. The DNAJB1-A2 chimera restored the refolding activity to the same extent as WT DNAJB1, while the DNAJA2-B1 chimera was inactive. Data in panels *A*, *E*, and *G* represent mean ± SD from three independent measurements.

We next asked whether the second major stress-inducible Hsp70, HSPA6 (9, 55), displays similar JDP selectivity. Fluorescence polarization assays of Alexa488-labeled JDP J-domains showed that, like HSPA1, HSPA6 interacts only weakly with the noncanonical Class B′ JDPs (K_D_s > 110 µM) (Fig. 4 *A* and *B* and *SI Appendix*, Table S1). Unexpectedly, HSPA6 showed very weak interaction with the canonical Class A J-domains, DNAJA1 and DNAJA2, two of the most conserved JDPs, which showed the strongest binding to both HSPA1 and HSPA8 (K_D_ values of 113 and 114 µM, respectively) (Fig. 4 *A* and *B* and *SI Appendix*, Table S1). The clear exceptions were the canonical Class B JDP J-domains, DNAJB1 and DNAJB5, which bound to HSPA6 with significantly higher affinities of 46 µM and 50 µM, respectively (Fig. 4 *A* and *B* and *SI Appendix*, Table S1). This clear binding preference was also visible in the 2D ^1^H-^15^N TROSY-HSQC NMR spectra of the DNAJB1 and DNAJA2 J-domains recorded in the presence and absence of unlabeled HSPA6 T205A. DNAJB1-HSPA6 showed a strong interaction, while only a very weak binding of HSPA6 to DNAJA2 was detected (Fig. 4 *C* and *D*). Thus, HSPA6 exhibits a unique and highly selective preference for canonical Class B JDPs, clearly distinguishing them from both Class A and noncanonical Class B′ JDPs.

Motivated by these striking differences in affinity, we next asked whether they translate into differences in functional cooperation with HSPA6. Based on our earlier observation that J-domain affinity aligns with refolding activity for HSPA1, we tested whether a similar relationship holds for HSPA6. To this end, we measured the refolding of chemically denatured FFL by HSPA6, in the presence of the full-length JDPs and a NEF (Hsp110). Remarkably, the canonical Class A JDPs, which had supported refolding with both HSPA1 and HSPA8, failed to do so with HSPA6. Only the canonical Class B JDPs, DNAJB1 and DNAJB5, which exhibited higher affinities, supported refolding activity by HSPA6 (Fig. 4*E*) To ensure that this lack of refolding activity is not due to the specific nature of the NEF, we repeated the refolding experiments with BAG1 and BAG3 nucleotide exchange factors. However, HSPA6 showed similarly highly reduced refolding activity with class A JDPs regardless of the nature of the NEF (Fig. 4*E* and *SI Appendix*, Fig. S6 *A* and *C*). Furthermore, we observed a strong correlation between the binding affinity of each J-domain for HSPA6 and its ability to support the Hsp70-dependent refolding activity with all three different nucleotide exchange factors (Fig. 4*F* and *SI Appendix*, Fig. S6 *B* and *D*).

To directly test whether the inability of DNAJA1 and DNAJA2 to support HSPA6-driven FFL refolding results from weak J-domain binding, we expressed and purified two chimeras - DNAJB1-A2 in which the high-affinity J-domain of DNAJB1 was fused to the client binding domains of DNAJA2, and DNAJA1-B1, in which the low-affinity J-domain of DNAJA2 was fused to the client binding domains of DNAJB1 (Fig. 4*G*). We then measured the ability of DNAJB1-A2 chimera to cooperate with HSPA6 in refolding denatured FFL and compared its activity to that of WT DNAJA2 and DNAJB1 (Fig. 4*G*). Excitingly, the DNAJB1-A2 chimera fully restored HSPA6-mediated refolding activity and performed similarly to DNAJB1, while the DNAJA2-B1 chimera showed very minimal refolding activity (Fig. 4*G*). These results confirm that the affinity of the J-domain alone is what dictates the degree of functional cooperation with HSPA6 and reinforce the notion that high-affinity JDP–Hsp70 interactions are essential for chaperone activity.

### The Plasticity of the J-Domain Loop Influences Its Affinity Toward HSPA6.

Since HSPA6 exhibits high selectivity and robust refolding activity with canonical Class B J-domains, while uniquely displaying low affinity for Class A J-domains, we aimed to uncover the structural basis underlying this selective interaction.

To do so, we first mapped the interaction of HSPA6 with each J-domain using NMR spectroscopy. 2D ^1^H-^15^N TROSY-HSQC spectra were recorded for all six J-domains in the presence and absence of unlabeled HSPA6 T205A (*SI Appendix*, Fig. S7). Mapping the intensity differences (I/I_0_) onto the J-domains structures revealed a conserved interaction pattern across most JDPs, centered at the end of helix II, the flexible loop containing the conserved HPD motif, and helix III. Interestingly, this conserved interaction through helix II was lost in Class A J-domains (*SI Appendix*, Fig. S7 *A* and *B*). Since helix II of the J-domains is known to interact with the NBD and the interdomain linker of Hsp70 (34), its absence could explain the weak binding and poor activation of HSPA6 by Class A JDPs.

**Fig. 5.**
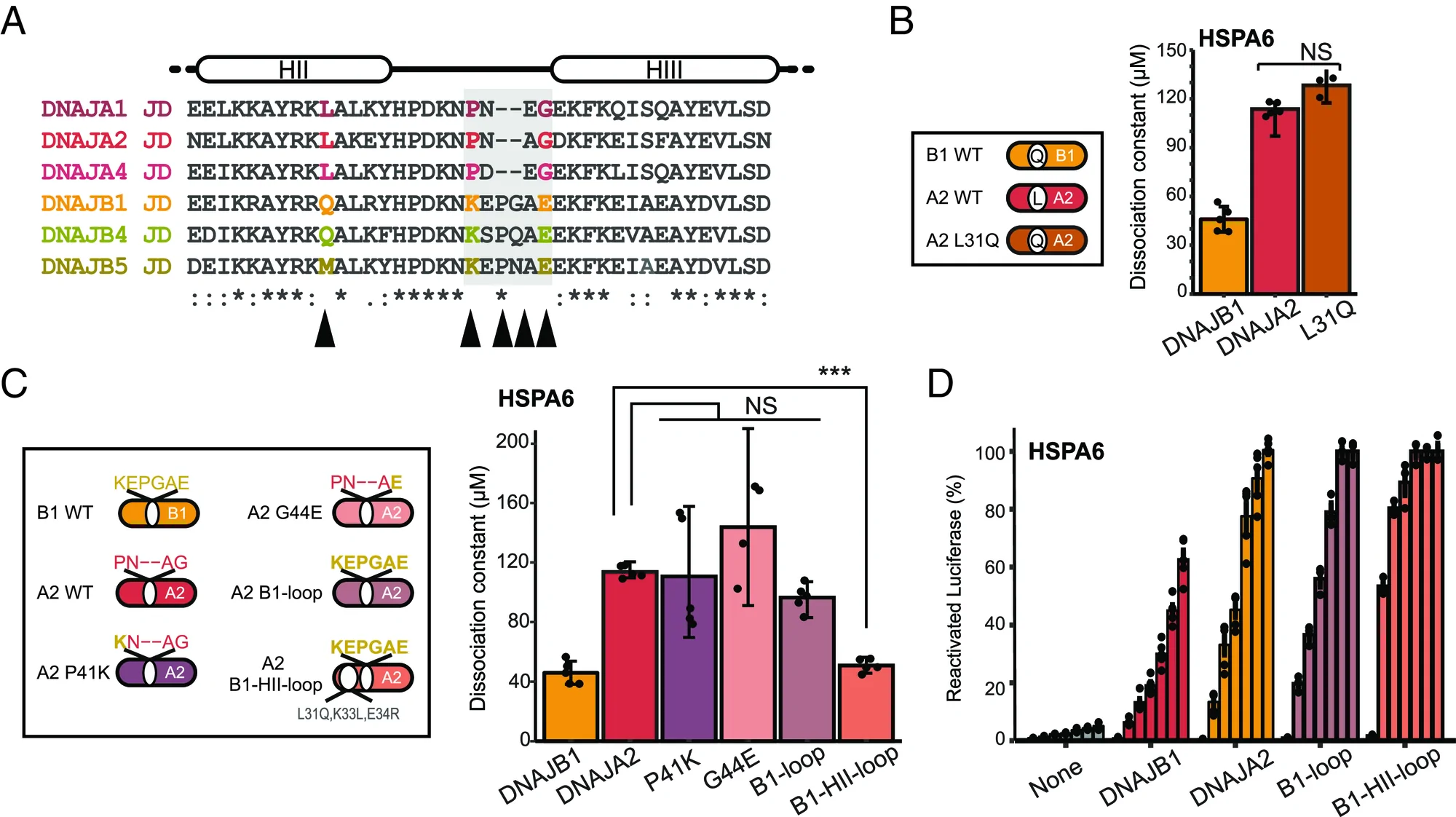
Sequence determinants of HSPA6 selective recognition of Class B J-domains. (*A*) Clustal Omega sequence alignment of representative J-domains, highlighting residues identified by NMR as part of the HSPA6-binding surface. Positions that explain affinity differences toward HSPA6 are shown in bold and color. The locations of helices II and III are indicated above the alignment. (*B*) Bar plots showing the dissociation constants (KD) for HSPA6 binding to DNAJB1 JD (yellow), DNAJA2 JD (red), and DNAJA2 L31Q JD (brown). (*C*) Bar plots showing the dissociation constants (KD) for HSPA6 binding to DNAJB1 JD (yellow), DNAJA2 JD (red), DNAJA2 P41K (purple), DNAJA2 G44E (pink), DNAJA2 B1-loop (lilac), and DNAJA2-B1-HII-loop chimera (coral). Individual data points represent independent fluorescence polarization titrations for 3 to 5 independent measurements. Bars indicate the mean KD, and error bars represent the corresponding 95% CI calculated from log-transformed KD values. Statistically significant differences for panels *B* and *C* are indicated with an asterisk (one-way ANOVA with Tukey–Kramer post hoc test). (*D*) Refolding activity of FFL mediated by HSPA6 and HSP110, in the absence (gray) or presence of WT DNAJA2 (red), WT DNAJB1 (yellow), DNAJA2 B1-loop chimera (lilac), and DNAJA2-B1-HII-loop variant (coral). FFL activity was measured at 0 min, 10 min, 20 min, 30 min, 60 min, 90 min, and 120 min. Data points represent the mean ± SD of three independent measurements.

To explore the molecular basis for this loss of interaction, we performed a sequence alignment specifically focusing on the J-domain residues that showed differential binding between Class A and B in our NMR experiments (Fig. 5*A*). While overall this region is highly conserved, a single nonconserved residue located at the end of helix II was identified that may explain the differential affinity between these classes (Fig. 5*A*). To test the importance of this position, we mutated DNAJA2 J-domain by replacing L31 with glutamine (L31Q), mimicking the sequence in DNAJB1. Fluorescence polarization measurements, however, showed that the L31Q mutation alone was not sufficient to alter the affinity toward HSPA6 compared to WT DNAJA2 J-domain (Fig. 5*B*).

In addition to this residue, we noticed distinct differences in the loop between helices II and III. To test whether these loop features modulate affinity for Hsp70, we generated several DNAJA2 variants in which the Class A residues were swapped with those from Class B. Specifically the following variants were generated: DNAJA2 P41K, DNAJA2 G44E, DNAJA2-B1-loop in which residues P41-D45 of DNAJA2 were swapped with those from the DNAJB1, and HII-loop DNAJB1 variant that also contained L31Q, K33L, E34R mutations in helix II making it highly homologous to DNAJB1 (DNAJA2-B1-HII-loop) (Fig. 5 *C*, *Left*). All the J-domain variants were purified, fluorescently labeled with Alexa488 fluorophore, and tested by fluorescence polarization for HSPA6 binding in comparison to WT DNAJB1 and DNAJA2.

Strikingly, the DNAJA2-B1-HII-loop variant showed a significant increase in affinity (51 µM), approaching that of DNAJB1 itself (46 µM) (Fig. 5*C* and *SI Appendix*, Tables S1 and S2). Individual point mutations within the loop (P41K and G44E) failed to improve the binding, while the variant that contained only the replacement of the loop residues (DNAJA2-B1-loop) showed a moderate improvement (96 µM) (*SI Appendix*, Table S2). This suggests that the overall sequence and flexibility of the loop as well as the composition of J-domain helix II, rather than individual specific residues, are critical for modulating affinity. Furthermore, we find that the loop length, charge, and structural flexibility play a role in tuning the J-domain affinity to Hsp70s, specifically in the case of the HSPA6 paralog.

Given our earlier observation that J-domain–Hsp70 affinity correlates with refolding activity, we next tested whether the improved binding of the loop-swapped DNAJA2 translated into enhanced functional activity. To this end, we generated two full-length DNAJA2 chimeras, one containing the DNAJB1-loop substitution, and the second DNAJA2-B1-HII-loop variant that included the DNAJB1-loop along with three additional mutations (L31Q, K33L, E34R) in helix II, matching the helix II sequence of DNAJB1. We measured the ability of both variants to refold chemically denatured FFL, alongside WT DNAJA2 and DNAJB1. Remarkably, the DNAJA2-B1-HII-loop variant fully restored Hsp70-dependent luciferase refolding activity and even outperformed WT DNAJB1 (reaching 100% refolding in less than an hour) (Fig. 5*D*), indicating that even subtle alterations in the conserved J-domain can tune chaperone function. Moreover, these results show that the flexible loop within the J-domain contributes significantly to Hsp70 paralog specificity and functional output.

### HSPA6–Class B JDP Selectivity Tunes and Diversifies JDP Functions under Acute Stress.

Based on our results, HSPA6 emerges as a unique Hsp70 paralog with pronounced selectivity for canonical Class B JDPs and markedly reduced affinity for canonical Class A JDPs, the most evolutionarily conserved cochaperones. This highly selective binding profile suggests a functional divergence of HSPA6 from other cytosolic Hsp70s.

**Fig. 6.**
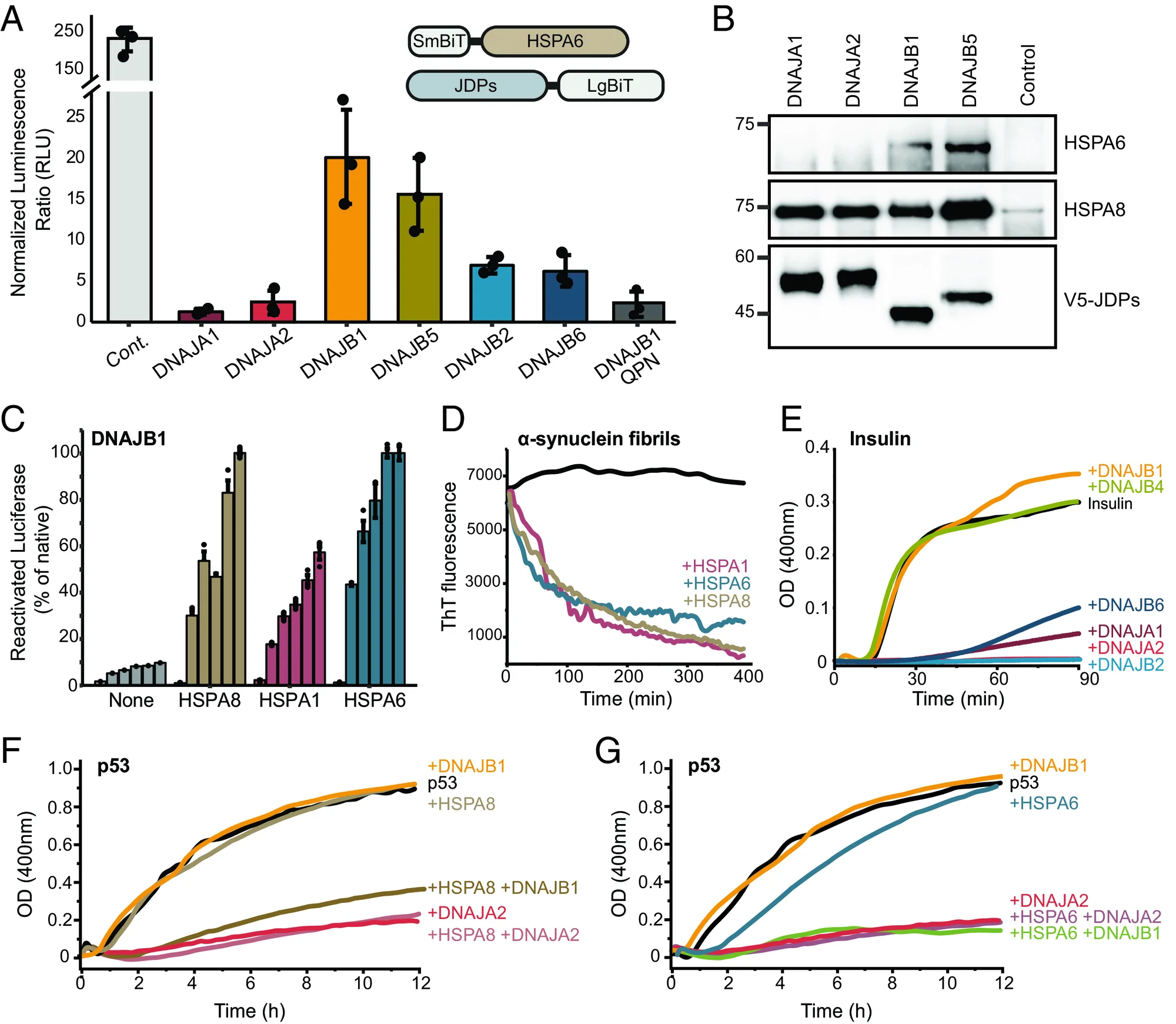
HSPA6 functions are determined by its affinity toward the J-domains. (*A*) Analysis of HSPA6–JDP interactions in cells using the NanoBiT protein complementation assay. HEK293T cells were cotransfected with a HSPA6-SmBiT and the individual JDP-LgBiT fusion constructs. Results are presented as normalized luminescence ratios (RLU) relative to the negative empty controls. DNAJB1-SmBiT and DNAJB1-LgBiT coexpression was used as a positive control, monitoring DNAJB1 homodimerization. DNAJB1 variant lacking the Hsp70-binding HPD motif (DNAJB1-QPN) (23, 58) served as a negative control. HSPA6 showed strong interactions with canonical Class B JDPs (DNAJB1 and DNAJB5), weaker interactions with noncanonical Class B′ JDPs (DNAJB2 and DNAJB6), and no detectable interaction with canonical Class A JDPs (DNAJA1 and DNAJA2). Data are representative of three independent experiments performed in triplicate. A schematic of the NanoBiT assay is shown above the graph. (*B*) Pull-down assays using V5-tagged DNAJA1, DNAJA2, DNAJB1, and DNAJB5 expressed in HEK293T cells under heat-shock conditions. Immunoprecipitation was performed using anti-V5 beads, and coprecipitated endogenous HSPA6 and HSPA8 were detected by western blotting. HSPA6 was efficiently coprecipitated only by the canonical Class B JDPs DNAJB1 and DNAJB5, whereas HSPA8 interacted with all tested JDPs. GFP-HSPA6 was used as a negative control. (*C*) Refolding activity of FFL mediated by DNAJB1, Hsp110, and the indicated Hsp70 chaperones—HSPA8 (brown), HSPA1 (pink), or HSPA6 (blue). (*D*) Disaggregation of α-synuclein fibrils by HSPA8 (brown), HSPA1 (pink), and HSPA6 (blue) with DNAJB1 and HSP110 included in all reactions as the JDP and NEF. The decrease in thioflavin T (ThT) fluorescence indicates the disaggregation of α-synuclein fibrils. (*E*) Light scattering measurements (OD 400 nm) following aggregation of insulin alone (black) (40 μM) or in the presence of twofold molar excess of indicated JDPs. Class B JDPs, DNAJB1 and DNAJB4 show no aggregation prevention activity toward insulin, while Class A and B′ JDPs fully suppress insulin aggregation. (*F*) Aggregation of destabilized p53 R249S monitored alone (black) or in the presence of a twofold molar excess of DNAJA2 (red), DNAJB1 (yellow), HSPA8 (brown), or mixtures of DNAJA2–HSPA8 (pink) or DNAJB1–HSPA8 (dark brown). (*G*) Aggregation of destabilized p53 R249S monitored alone (black) or in the presence of a twofold molar excess of DNAJA2 (red), DNAJB1 (yellow), HSPA6 (blue) or mixtures of DNAJA2–HSPA6 (purple) or DNAJB1–HSPA6 (green). Data in panels *C*–*G* represent mean ± SD from three independent measurements.

To determine whether these interaction patterns are maintained in a cellular context, we employed a NanoBiT protein complementation assay in HEK cells (56, 57) (Fig. 6*A*). HSPA6 exhibited strong and selective interactions with canonical Class B JDPs (DNAJB1 and DNJAB5), whereas interactions with the noncanonical Class B′ JDPs (DNAJB2 and DNAJB6) were markedly weaker (Fig. 6*A*). No detectable interaction was detected between HSPA6 and Class A JDPs (DNAJA1 and DNAJA2) (Fig. 6*A*). These cellular results are in excellent agreement with our in vitro measurements and demonstrate that the intrinsic binding preferences of HSPA6 paralog are preserved in the cellular environment. Moreover, when considered in the context of reported intracellular protein concentrations (*SI Appendix*, Table S3) (42), the very low affinity of HSPA6 for Class A JDPs strongly suggests that such interactions are effectively excluded under physiological conditions.

As HSPA6 is robustly induced only under prolonged proteotoxic stress (9, 55) (*SI Appendix*, Fig. S8), we next tested whether these interaction preferences are maintained for endogenously expressed Hsp70s under stress conditions. To this end, we performed pull-down experiments using V5-tagged Class A (DNAJA1 and DNAJA2) and Class B (DNAJB1 and DNAJB5) JDPs as bait. HEK cells were exposed to 42 °C heat shock for 60 min to induce stress and were collected after 20 h, under which HSPA6 expression is strongly upregulated (*SI Appendix*, Fig. S8*C*). The use of V5-tagged constructs ensured comparable expression levels of all four JDPs (Fig. 6*B*), minimizing bias arising from stress-induced upregulation of endogenous DNAJB1 (*SI Appendix*, Fig. S8*D*). Under these conditions, only Class B JDPs, DNAJB1, and DNAJB5 efficiently coprecipitated HSPA6, whereas Class A JDPs did not. In contrast, HSPA8 was readily pulled down by all tested JDPs at comparable levels (Fig. 6*B*).

Together, these results demonstrate that HSPA6 activity in cells is selectively restricted to cooperation with Class B JDPs, thereby tuning JDP function under conditions of acute proteotoxic stress.

### A dedicated HSPA6–Class B JDP Module Promotes Proteostasis under Stress.

Having established that HSPA6 selectively interacts with Class B JDPs in cells, we next asked whether these JDPs can function as bona fide HSPA6 cochaperones capable of supporting the full range of Hsp70-dependent activities, including stimulation of ATP hydrolysis and assistance in protein folding and refolding. Indeed, all Class B JDPs tested (DNAJB1, DNAJB4, and DNAJB5) enhanced HSPA6 ATPase activity by more than 4.5-fold. In contrast, Class A JDPs showed only a very moderate HSPA6 activation (*SI Appendix*, Fig. S8*E*), despite stimulating HSPA8 and HSPA1 ATPase activity to a similar extent as Class B JDPs (*SI Appendix*, Fig. S1*B*-*C*). Consistent with this, HSPA6 displayed highly efficient luciferase refolding in the presence of DNAJB1 and DNAJB5, with refolding kinetics considerably faster than those mediated by either HSPA1 or HSPA8 (Fig. 6*C* and *SI Appendix*, Fig. S8*F*), suggesting that the HSPA6-Class B JDP module provides even superior refolding capacity compared to other Hsp70 paralogs.

One of the most unique functions of Class B JDPs is their ability to disaggregate preformed amyloid fibers in collaboration with HSPA8 and a specific NEF, Hsp110 (21, 22, 59, 60). We therefore examined whether HSPA6 can support similar activity. To this end, we have tested the ability of DNAJB1, HSPA6, and Hsp110 to disaggregate preformed α-synuclein fibrils and compared the activity to that of DNAJB1 and Hsp110 with HSPA1 and HSPA8. We found that HSPA6 is indeed capable of mediating α-synuclein disaggregation as efficiently as HSPA8 and HSPA1 (Fig. 6*D*). Thus, Class B JDPs can function as HSPA6 cochaperones, fully supporting all Hsp70 stress-related activities. Interestingly DNAJB1 levels are also strongly upregulated under stress (6, 55) (*SI Appendix* Fig. S8*D* and Table S3), which suggests that under severe stress DNAJB1, together with HSPA6, form a dedicated chaperone module optimized for rapid proteostasis recovery through ATP-driven refolding and disaggregation.

While Class B JDPs fully support Hsp70-dependent functions with HSPA6, the question remains why HSPA6 has lost the ability to form productive complexes with Class A JDPs. Class A JDPs, similarly to the noncanonical Class B′ JDPs (26, 27, 61), efficiently suppress aggregation of many misfolded and aggregation prone-client proteins (19, 20, 62, 63) (Fig. 6*E* and *SI* *Appendix*, Fig. S8*G*) and were recently reported to sequester β-sheet–rich proteins on the verge of misfolding and protect them from further misfolding and aggregation (19). Remarkably, in contrast, Class B JDPs displayed little to no Hsp70-independent anti-aggregation activity toward any of the substrates we tested (19) (Fig. 6*E* and *SI Appendix*, Fig. S8*G*). Thus, whereas Class A and Class B′ JDPs can function in an Hsp70-independent manner to prevent protein aggregation, a function that is particularly critical during stress, Class B JDPs do not operate as stand-alone chaperones but rather as components of an Hsp70-dependent module.

This distinction became clear when the p53 client protein was incubated with mixtures of DNAJB1 and Hsp70s. In the absence of Hsp70, DNAJA2 showed efficient aggregation prevention activity, while DNAJB1 was completely inactive. However, in the presence of Hsp70s, specifically HSPA6, a highly efficient aggregation prevention activity was detected for DNAJB1, comparable to that obtained with DNAJA2 (Fig. 6 *F* and *G*). Thus, Class B JDPs do not function as stand-alone holdases but rather as components of an Hsp70-dependent module that requires Hsp70 engagement to become effective.

**Fig. 7.**
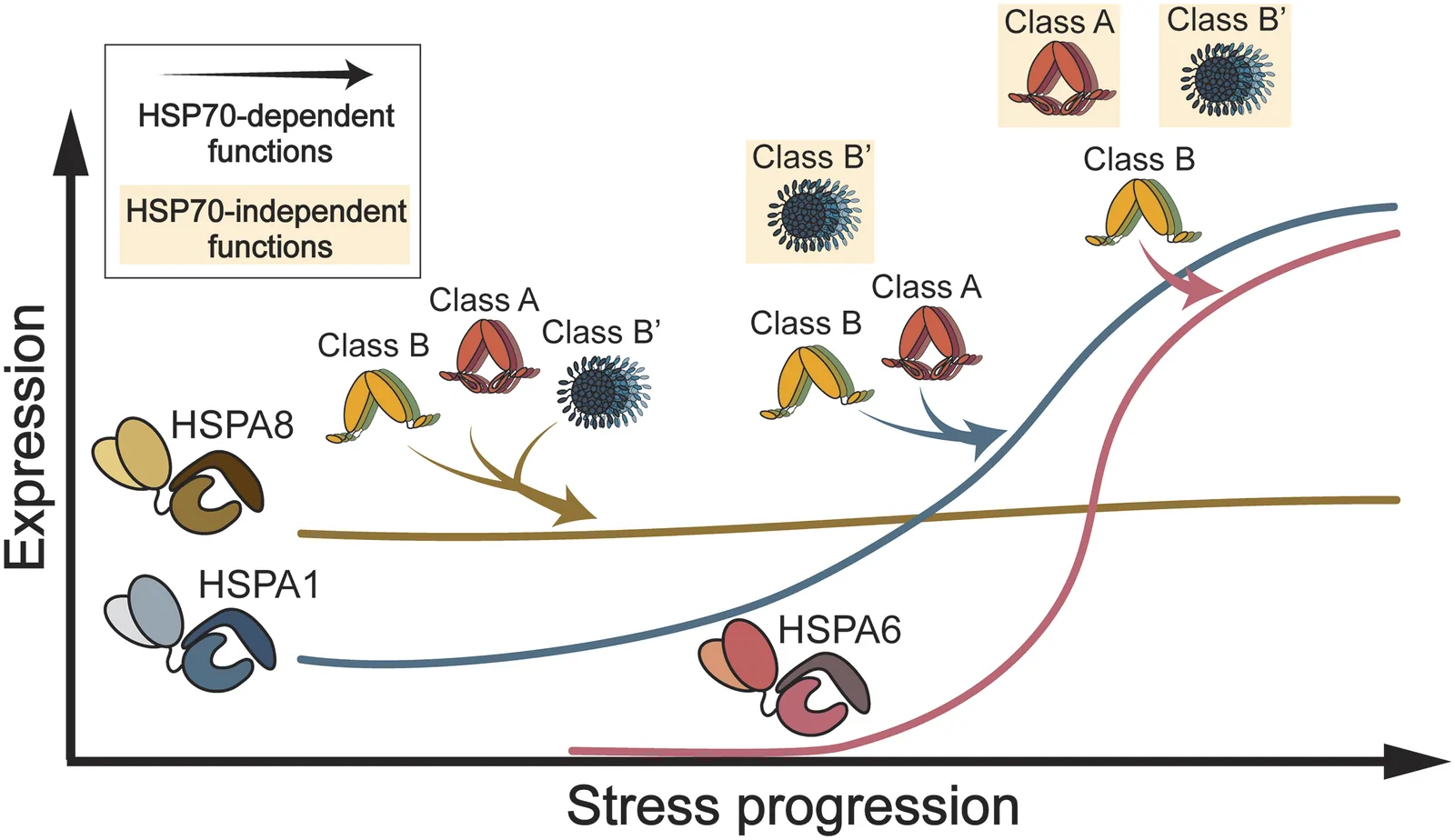
Stress-driven switch from Hsp70-dependent repair to protection. Model displaying how Hsp70 paralog redistributes interactions with Class A, B, and B′ JDPs as a function of cellular stress progression. Under normal, unstressed conditions, the constitutively expressed HSPA8 engages broad-specificity JDPs from classes A, B, and B′, thereby supporting the full set of Hsp70-dependent functions mediated by these cochaperones. As stress increases, stress-inducible HSPA1 becomes upregulated. HSPA1 preferentially binds the canonical Class A and Class B JDPs, releasing the noncanonical Class B′ JDPs to function independently of Hsp70—primarily by preventing client misfolding and aggregation. Under severe stress, the levels of stress-inducible HSPA6 are markedly increased. HSPA6 selectively binds canonical Class B JDPs, enabling HSPA6-Class B JDPs complexes to sustain essential Hsp70-dependent activities, while freeing both Class A and Class B′ JDPs to act independently of Hsp70 preventing protein misfolding and aggregation. Together, the stress-driven hierarchy of Hsp70–JDP affinity preferences shifts the cellular response from Hsp70-dependent repair toward Hsp70-independent protective pathways.

Such selective coupling between HSPA6 and Class B JDPs not only maximizes Hsp70-dependent repair activities but also ensures that generalist Class A and B′ JDPs, with strong Hsp70-independent functions, remain free to operate in parallel pathways, such as aggregation prevention and sequestration of misfolded proteins. Together, our findings support a model in which HSPA6 selectively engages with canonical Class B JDPs to maintain essential Hsp70-dependent activities during acute proteotoxic stress, while allowing other JDP classes to provide complementary, Hsp70-independent protection (Fig. 7).

## Discussion

Cytosolic Hsp70 chaperones, together with their JDP cochaperones, play a key role in regulating proteostasis by mediating protein folding, refolding, assembly, degradation, and aggregation-prevention (1–4). However, little was known about the distinct functions of the cytosolic Hsp70s and their specific interactions with the various JDP family members in humans.

Our work reveals that cytosolic Hsp70 paralogs engage JDPs through a hierarchy of paralog-selective interactions that partition proteostasis into parallel, functionally specialized pathways prioritized according to stress level. Although interaction between Hsp70s and the JDP J-domain is known to be highly conserved (17, 64), our data show that subtle sequence and structural differences within the conserved J-domain create surprisingly strong biases in binding affinity. These differences vary across JDP classes and Hsp70 paralogs, making Hsp70 paralog identity a key predictor of JDP pairing and, consequently, chaperone function during stress.

Interestingly, while the stress-induced Hsp70s (HSPA1 and HSPA6) show strong and selective preferences for different JDP classes, the constitutively expressed HSPA8 (Hsc70) exhibits similar affinities toward all examined Class A, Class B, and noncanonical Class B′ JDPs. This broad binding spectrum is consistent with HSPA8 serving as the primary housekeeping Hsp70, responsible for de novo protein folding, remodeling of protein complexes, chaperone-mediated autophagy, as well as for recruiting substrates to the proteasome (65–67). Because each of these pathways relies on different JDP partners, HSPA8 must engage all of them productively. Its equal affinity for broad-specificity JDPs ensures precisely that, enabling HSPA8 to maintain essential cellular functions under basal conditions and to continue refolding, aggregation prevention, and disaggregation under stress.

One of the unexpected findings is that while canonical Class A and Class B JDPs robustly support Hsp70-dependent refolding of model substrates, the noncanonical Class B′ JDPs (DNAJB2 and DNAJB6) exhibit minimal refolding activity under identical conditions. Class B′ JDPs evolved later and diverged substantially from canonical JDPs in structure and function (68), particularly within their CTD client-binding domains. Our chimera experiments demonstrate that the inability of DNAJB2 and DNAJB6 to support refolding is entirely determined by the nature of these domains, as replacement with the DNAJA2 client-binding domains fully restored HSPA8-dependent refolding activity (Fig. 2*E*). These results, in line with the low upregulation of Class B’ JDPs during stress, suggest that Class B′ JDPs are not optimized for Hsp70-mediated protein refolding, but instead may aid Hsp70 in an array of housekeeping functions under nonstress conditions. Consistent with this, DNAJB2 was reported to promote degradation of ubiquitinated proteins through its ubiquitin-interacting motifs (66, 69), and DNAJB6 to support phagophore formation, autophagy-related pathways, and nuclear pore complex biogenesis (70–73).

Importantly, our data suggest that under stress conditions Class B′ JDPs are unlikely to form substantial complexes with HSPA1. Given reported intracellular abundances (*SI Appendix*, Table S3) and stress-induced expression levels (41, 42), the relatively weak interactions observed for DNAJB2 and DNAJB6 with HSPA1 (K_D_s ≥ 65 µM) are unlikely to be significantly populated at steady state. In the absence of productive interactions with Hsp70 partners, these JDPs instead act as stand-alone chaperones. This independent function is particularly important during stress, when HSPA1 levels are upregulated, given both their inability to support Hsp70-dependent refolding (Fig. 2 *A* and *B*) and their potent antiaggregation activity (25–28, 30, 70, 73). Thus, while Class A and Class B JDPs support HSPA1-dependent protein (re)folding and disaggregation, Class B′ JDPs likely act as a buffering system that maintains proteins in a non-aggregated state until cellular refolding capacity is restored.

Under severe stress conditions, the stress-inducible HSPA6, which lacks basal expression, is strongly upregulated (9, 55). Unexpectedly, we find that HSPA6 exhibits very low affinity for Class A JDPs (K_D_ > 110 µM), the most evolutionarily conserved Hsp70 cochaperones, as well as to the noncanonical Class B′. As a result, even under conditions of robust HSPA6 induction (e.g., under severe and prolonged stress), we find these JDPs do not interact with HSPA6 in cells (Fig. 6 *A* and *B* and *SI Appendix*, Fig. S8). Consequently, both Class A and Class B′ JDPs are likely to function largely in an Hsp70-independent manner during severe stress, preventing protein misfolding and aggregation across a broad range of substrates (Fig. 6*E* and *SI Appendix*, Fig. S8*G*) (19, 30, 61). Thus, HSPA6 emerges as a unique Hsp70 that interacts exclusively with Class B JDPs. This is consistent with proteomic analyses showing HSPA6 association (by AP-MS) only with members of the Class B family, whereas HSPA1 interacts with both Class A and Class B JDPs (74).

Importantly, both HSPA6 and Class B DNAJB1 are strongly induced during acute stress (*SI Appendix*, Fig. S8*D* and Table S3) (6, 75, 76), creating a highly specific and dedicated HSPA6-Class B module that channels all ATP-driven Hsp70-dependent refolding and disaggregation activities through a single axis. In parallel, the very low affinity of HSPA6 for both Class A and Class B′ JDPs prevents the formation of futile, ATP-consuming Hsp70 cycles while leaving these chaperones available to function independently of HSPA6 and suppress protein aggregation, a function that is particularly important during acute stress to prevent irreversible proteotoxic damage.

We therefore propose that, during cellular stress, the chaperone network is dynamically rewired through paralog-specific JDP interactions. During moderate stress, HSPA1 works with Class A and Class B JDPs to ensure protein repair (refolding and disaggregation), while Class B′ JDPs act independently to suppress aggregation (19, 30, 62). In parallel, when stress is prolonged/severe, HSPA6 is strongly induced and selectively couples to Class B JDPs, which cannot function as stand-alone chaperones, to prevent protein aggregation (Fig. 6 and *SI Appendix*, Fig. S8) (77, 78). This exclusive HSPA6–Class B pairing creates a dedicated, high-throughput repair module that accelerates ATP-driven refolding and disaggregation, with both Class A and Class B′ JDPs performing Hsp70-independent aggregation suppression and sequestration protection functions in parallel (Fig. 7). Thus, in response to stress, paralog-specific JDP interactions rewire Hsp70 functions, progressively shifting the balance from an Hsp70-dependent repair focused module, responsible for (re)folding and disaggregation, to an Hsp70-independent containment module that prevents protein misfolding and aggregation.

Collectively, our findings challenge the notion that Hsp70 paralogs are interchangeable. By identifying sequence-encoded features that tune paralog selectivity, we reveal how conserved J-domains have been evolutionarily refined to redistribute JDPs between Hsp70-dependent and independent pathways as a function of cellular stress to ensure robust protein homeostasis.

## Materials and Methods

Human cytosolic Hsp70 paralogs, JDPs, NEFs, client proteins, engineered variants, and chimeric constructs were recombinantly expressed and purified from *Escherichia coli*. Mammalian expression constructs were used for NanoBiT interaction assays, coimmunoprecipitation experiments, and heat-stress studies in HEK293 cells. Detailed descriptions of plasmids, construct generation, protein purification procedures, labeling strategies, and cell-based assays are provided in *SI Appendix*.

Hsp70–JDP interactions were quantified by fluorescence polarization using Alexa488-labeled J-domains (50 nM) and increasing concentrations of Hsp70 proteins (HSPA1^T204A^, HSPA6^T205^, HSPA8^WT^, or HSPA8^T204A^) in the presence of ATP or ADP. Each titration was fit independently to a one-site binding model, and dissociation constants (K_D_) are reported as geometric means with corresponding 95% CI calculated from log-transformed K_D_ values. For graphical representation, fluorescence polarization curves were normalized using min–max normalization. Detailed experimental conditions and data analysis procedures are provided in *SI Appendix*.

Chaperone activity was assessed primarily using FFL refolding assays. Chemically denatured luciferase was diluted into refolding reactions containing the indicated Hsp70 paralog, full-length JDP, nucleotide exchange factor (Hsp110, BAG1, or BAG3), and an ATP regeneration system. Refolding was monitored by recovery of luciferase activity over time and expressed relative to maximal refolding yields. For detailed method description, please see *SI Appendix*. Additional chaperone activities were evaluated using ATPase assays, citrate synthase refolding assays, α-synuclein fibril disaggregation assays, and aggregation prevention assays with insulin, p53 R249S, and TDP-43, as described in *SI Appendix*.

Solution NMR experiments were performed on Bruker spectrometers operating at magnetic field strengths of 14.1 T and 23.5 T. Binding interfaces were identified from changes in peak intensities in ^1^H–^15^N TROSY-HSQC spectra acquired for ^15^N-labeled J-domains in the absence and presence of Hsp70 proteins. Backbone resonance assignments for the DNAJB5 J-domain, resulting in unambiguous assignments of 99%, nonproline residues, are described in *SI Appendix*.

All quantified experiments were performed with at least three independent replicates. Data are presented as mean ± SD, and statistical significance was assessed using one-way ANOVA with Tukey–Kramer post hoc analysis. Detailed descriptions of all experimental conditions and data analysis are provided in *SI Appendix*.

## Supplementary Material

Appendix 01 (PDF)

## Data Availability

Raw data are available online at (79) https://doi.org/10.34933/6446094f-5b2e-46f6-86c2-181f48c15136. NMR chemical shifts for DNAJB5^JD^ have been deposited in the Biological Magnetic Resonance Data Bank (BMRB) under the accession code 53438. All other data are included in the manuscript and/or *SI Appendix*.
