# Supplementary material for: Human Hsp70 paralogs display selective J-domain interactions tuning proteostasis under stress
Source: Proc Natl Acad Sci U S A. 2026 Aug 18;123(34):e2534903123. doi: 10.1073/pnas.2534903123 (PMC13506082; doi:10.1073/pnas.2534903123)
Supplement: Supplementary file 1 [file pnas.2534903123.sapp.pdf]

Supporting Information for

Human Hsp70 paralogs display selective J-domain interactions tuning proteostasis under stress

Roni Suhler[1], Lars J.W. van Beurden[1], Merav Shmueli[2], Ofrah Faust[1], and Rina Rosenzweig[1,*].

[1]Department of Chemical and Structural Biology, Weizmann Institute of Science, Rehovot, Israel

[2]Department of Systems Immunology, Weizmann Institute of Science, Rehovot, Israel

*Rina Rosenzweig

Email: rina.rosenzweig@weizmann.ac.il

This PDF file includes:

Supporting text

Figures S1 to S8

Tables S1 to S3

Materials and Methods

Plasmids and constructs preparation

HSPA8, HSPA1, HSPA6, DNAJB1 and the chimeric DNAJB1-A2 and DNAJA2-B1 construct (DNAJB1 residues 1-76 fused to DNAJA2 residues 79-412, and DNAJA2 residues 1-77 were fused to DNAJB1 residues 79-340) with an N-terminal His_6-sumo tag, were a generous gift from the Bukau lab (University of Heidelberg). BAG1 construct was a generous gift from the Wentink lab (Leiden University). The ATPase-deficient $HSPA8^{T204A}$, $HSPA1^{T204}$, $HSPA1^{T204,K423E}$, and $HSPA6^{T205}$ mutants, and $HSPA1^{K423E}$ were created using QuickChange mutagenesis.

Codon optimized DNAJA1 and DNAJA2 and BAG3 were expressed from pET-29b(+) (Kan^R) vector with an N-terminal His_6 tag followed by a tobacco etch virus (TEV) protease cleavage site. Codon optimized DNAJB2, DNAJB4, DNAJB5 and DNAJB6 were expressed from a pET-29b(+) (Kan^R) vector with an N-terminal His_6-sumo purification tag. The chimeric DNAJA2-B2 construct (DNAJA2 residues 79-412 fused to DNAJB2 residues 1-72) and DNAJA2-B6 construct (DNAJB6 residues 1-71 fused to DNAJA2 residues 79-412) constructs were created by restriction-free (RF) cloning. DNAJA1 JD (1–69), DNAJA2 JD (1–77), DNAJB1 JD (1–71), DNAJB2 JD (1-72), DNAJB5 JD (1-76) and DNAJB6 JD (1–70) constructs were created by introducing a stop-codon after the J-domain by Transfer-PCR (TPCR). The J-domain constructs for fluorescence polarization were created by introducing an additional C-terminal cysteine amino acid prior to a stop codon to the above constructs. All additional mutations to the various constructs were introduced by QuickChange or TPCR.

Plasmids encoding for TDP-43 with a N-terminal MBP-His_6 tag and a TEV cleavage site, and phosphate binding protein (PBP) with A197C mutation were acquired from Addgene (Addgene plasmid #104480; http://n2t.net/addgene: 104480; RRID: Addgene_104480 and Addgene plasmid #78198; http://n2t.net/addgene: 78198; RRID: Addgene_78198). p53 DBD wt and mutants in pET27 vector were a generous gift from Zippora Shakked (Weizmann Institute of Science). Citrate synthase with a N-terminal His_6 followed by a TEV cleavage site was a generous gift from Katrin Ulrich (University of Cologne). All additional mutations to the various constructs were introduced by QuickChange or TPCR.

Plasmids encoding for V5-tagged DNAJA1, DNAJA2, DNAJB1, and DNAJB5 as well as GFP-tagged HSPA6, in the pcDNA5 backbone were obtained from Addgene (Addgene plasmid # 19518; http://n2t.net/addgene:19518; RRID: Addgene_19518, Addgene plasmid # 19519; http://n2t.net/addgene:19519; RRID: Addgene_19519, Addgene plasmid # 19522; http://n2t.net/addgene:19522; RRID: Addgene_19522, Addgene plasmid # 19527; http://n2t.net/addgene:19527; RRID: Addgene_19527 and Addgene plasmid # 19486; http://n2t.net/addgene:19486; RRID: Addgene_19486).

NanoBiT vectors 11S-C, 11S-N, 114-C, and 114-N cloned into pCI-neo mammalian expression vectors under the control of a CMV enhancer/promoter were generous gifts from the Bajorek group (INRAE, France). For NanoBiT assays, HSPA6 was fused to the N-terminus of the NanoBiT small fragment (SmBiT), whereas all JDPs were fused to the C-terminus of the NanoBiT large fragment (LgBiT) via a GGGSGGSSGGS linker. The JDP fusion constructs 11S-C DNAJA1, 11S-C DNAJA2, 11S-C DNAJB1, 11S-C DNAJB2, and 11S-C DNAJB6 were provided by the Bajorek group, whereas 11S-C DNAJB5, 114-C DNAJB1, and 114-N HSPA6 were generated in this study using RF cloning or TPCR. Empty NanoBiT vectors were used as negative controls, and C-terminally tagged DNAJB1-SmBiT and DNAJB1-LgBiT constructs were used as positive controls.

Protein expression and purification

HSPA8 (1), HSPA1 (2), DNAJB1 (1), DNAJA1 (3), DNAJA2 (3), DNAJB6 (4), Hsp110 (1), PBP (5), FFL (6), citrate synthase (7), p53 R249S (3), and TDP-43 (8), BAG1 and BAG3 (9) were expressed and purified according to previously published protocols.

DNAJB2, DNAJB4, DNAJB5 and HSPA6 constructs were expressed in Escherichia coli BL21 (DE3) cells (Novagen). Cells were grown in Luria Bertani broth (LB) at 37°C to an OD600 of 0.8. Protein expression was induced by the addition of 1 mM isopropyl-β-D-thiogalactoside (IPTG) and incubation at 25°C or 18°C overnight. ^{15}N-labelled cells were grown with the same procedure but in M9 minimal media supplemented with $^{15}NH_4Cl$ as the only source of nitrogen. The cells were harvested by centrifugation and either frozen at -20°C or resuspended for purification.

Following harvesting, DNAJB2, DNAJB4, DNAJB5 cells were lysed by French Press in 50 mM Tris pH 8.0, 300 mM NaCl, 10 mM imidazole, protease inhibitor cocktail (Sigma), and DNase (Roche Diagnostics) and purified on a 5 ml HiTrap HP column (Cytiva). The proteins were released from the column with 50mM Tris pH 8.0, 300mM KCl, and 300 mM imidazole. The proteins were dialyzed overnight at 4°C, with the proper cleavage enzyme (TEV or Ulp1 for sumo constructs) to remove the His_6-tag. The cleaved proteins were further purified on a Ni-NTA column to separate them from the cleavage enzymes and uncleaved protein fractions, and further purified on a size-exclusion column (Superdex 75 pg 16/600 or Superdex 200 pg 16/600 (Cytiva) pre-equilibrated with 50mM HEPES pH 7.5, 150mM KCl 2mM DTT. The eluted fractions were aliquoted and snap-frozen in liquid nitrogen and stored at −80 °C. HSPA6 cells were lysed by French Press in 50 mM Tris pH 8.0, 300 mM NaCl, 10 mM imidazole, protease inhibitor cocktail (Sigma), and DNase (Roche Diagnostics) and purified on a 5 ml HisTrap HP column (Cytiva). The proteins were released from the column with 50mM Tris pH 8.0, 300mM KCl, and 300 mM imidazole. The His_6-tag was removed by an overnight cleavage with Ulp1 protease at 4 °C dialyzed into 50mM Tris pH 7, 150mM KCl, 10mM $MgCl_2$, 2mM DTT, 1mM ATP. The cleaved proteins were further purified on a Ni-NTA column to separate them from His-tagged Ulp1 and uncleaved protein fractions, and further purified on a size-exclusion Superdex 200 pg 16/600 (Cytiva) pre-equilibrated with 50mM Tris pH 7, 150mM KCl, 10mM $MgCl_2$, 2mM DTT, 1mM ATP. The eluted fractions were concentrated, aliquoted, snap-frozen in liquid nitrogen, and stored at −80 °C.

All NMR samples were in 50 mM HEPES pH 7.0, 50 mM KCl, 10% D2O, 0.03% NaN_3.

Labeling the proteins with fluorescent dyes

All J-domain proteins for fluorescence polarization were labeled with Alexa-488 C5-maleimide (Invitrogen) through the introduced C-terminal cysteine. Labelling was achieved by incubating the proteins with a 3-fold molar excess of Alexa Fluor 488 C5-maleimide for 30 min at 25°C or overnight at 4°C in 50 mM HEPES pH 7.0, 50 mM KCl. Excess of the fluorescent dye was removed using a desalting column. Labelling efficiencies were determined by LC/MS on a Waters ACQUITY UPLC class H instrument and the degree of labelling exceeded at least 90% in all cases.

Fluorescence polarization measurements

Steady-state equilibrium binding of Hsp70 chaperones ($HSPA1^{T204A}$, $HSPA6^{T205}$, $HSPA8^{WT}$, or $HSPA8^{T204A}$) and the J-domains were measured by fluorescence polarization, using 50 nM of fluorescently labeled proteins. All the experiments were done in 50 mM HEPES pH 7.0, 100 mM KCl, 10 mM $MgCl_2$, and in the presence of 2 mM ATP or ADP. The proteins were incubated for 20 min at 37 °C in a black flat-bottomed 384-square-well plate. Measurements were performed on a Synergy H1 microplate reader (BioTek). The excitation filter was centered on 485 nm with a bandwidth of 20 nm, and the emission filter was centered on 528 nm with a bandwidth of 25 nm. All fluorescence polarization assays were performed in triplicate. All

experiment replicates were fitted to a one-site binding model using OriginPro. Raw data from individual experimental replicates were fitted separately to a one-site binding model using OriginPro to extract K_D values. Reported dissociation constants correspond to the geometric mean of the independently determined K_D values, and 95% confidence intervals were calculated from the log-transformed K_D values. Final K_D values were calculated from at least three replicates.

For graphical representation, fluorescence polarization curves were normalized to a 0-1 scale using min–max normalization. Each value was rescaled relative to the minimum and maximum values within the dataset, such that the lowest value was set to 0 and the highest value to 1.

ATPase assay

Hsp70 phosphate-release rates after ATP hydrolysis were measured under steady-state conditions by monitoring the change in fluorescence of the phosphate-binding protein (PBP) A197C mutant, which was labelled at Cys197 using 7-diethylamino-3-[N-(4-maleimidoethyl)carbamoyl]coumarin (MDCC, CDX-D0198 from Adipogen).

All reactions contained PBP, 0.1 µM Hsp70, and 1 µM of indicated J-domains. The reaction was performed in 50 mM HEPES pH 7.0, 50 mM KCl, and 10 mM $MgCl_2$. After the plate was incubated at 37°C for 10 min, the reactions were started by injection of 100 µM ATP into each well. Fluorescence was measured by excitation at 430 nm and emission at 465 nm every 30 seconds for the first 20 minutes. A series of seven phosphate concentrations ranging between 0.25-1.5 µM was measured with PBP to generate a calibration curve, which was used to correlate fluorescence to the concentration of the released phosphate. The ATPase activity was determined by the initial rate of each reaction.

Luciferase refolding assay

Recombinant firefly luciferase (0.2 µM) was incubated for 40 min at 30 °C in denaturation buffer (25 mM HEPES-KOH, pH 7.5, 50 mM KCl, 10 mM MgCl2, 2 mM DTT, 6 M guanidinium chloride). To start the refolding reaction, the denatured luciferase was diluted 100-fold into refolding buffer (50 mM HEPES, pH 7.6, 50 mM KCl, 10 mM MgCl2, 2 mM DTT, and 0.1 mg/ml BSA). To initiate the refolding, 4 µM of Hsp70 (HSPA1, HSPA6 or HSPA8), 4 µM of indicated full-length JDPs, 0.4 µM NEF (Hsp110, BAG1, or BAG3) and ATP regeneration system were added. The ATP regeneration system was composed of Creatine Phosphokinase 50 U, ATP 5 mM and Phosphocreatine 20 mM. Luminescence was measured after 0, 10, 20, 30, 40, 60, 90 and 120 min by addition of 50 µM of luciferase reagent (Promega) to 9 µl of the refolding reaction in a white 96 well (Nunc) plate using a Synergy H1 plate reader (BioTek). Maximal refolding yields were set as 100%.

Citrate synthase refolding assay

Recombinant citrate synthase (25 µM) was denatured in denaturing buffer (6 M GnHCl, 50 mM HEPES pH 8, 50 mM KCl, 10 mM $MgCl_2$, 2mM EDTA) for 40 minutes at 30°C, 300 rpm. The refolding was initiated by diluting the denatured Citrate synthase 250-fold into the chaperone reactions containing 4 µM HSPA8, 4 µM of the full-length J-domain protein (DNAJA2, DNAJB1, DNAJB2, DNAJB6) and 0.4 µM of Hsp110 in 50 mM HEPES pH 8, 50 mM KCl, 10 mM $MgCl_2$, 2 mM ATP, 0.5 units/µl creatine kinase (CPK), and 25 mM phosphocreatine.

The reactions were incubated at 25°C, 300 rpm, and at indicated times (0, 10, 20, 30, 60, 90 and 120 min), 20 µl of the refolding mixture was added to a transparent, flat-bottomed, 96-well plate (Corning) preloaded with 130µl of refolding buffer (indicated above). After injection of 50µl reaction buffer containing 400 µM Oxaloacetic acid (Sigma Aldrich), 200 µM acetyl-CoA (Sigma Aldrich), 400 µM 5,5′-Dithiobis(2-nitrobenzoic acid) (Sigma Aldrich) in the refolding buffer described above into the refolding reaction, the absorbance at 412 nm was measured every 2 s for 1 minute using a Synergy H1 microplate reader (BioTek).

p53 aggregation prevention

p53 was purified as previously described. Aggregation of p53 R249S was monitored by measuring light scattering at 400nm in a buffer containing 50 mM NaPi pH 7.2, 150 mM NaCl. The aggregation assay was conducted at 37 °C with continuous shaking (567 rpm) and monitored at 400 nm in flat-bottom, clear, 96-well plates sealed with optical adhesive film (Applied Biosystems). Samples were run in replicates, and the experiments were repeated five times with similar results.

TDP-43 aggregation prevention assay

MBP-tagged TDP-43 (6 μM) was incubated in the presence or absence of indicated JDPs (12 μM) for 10 minutes at 37 °C. The aggregation was induced by cleavage of the MBP solubility tag with 0.3 μM TEV protease and monitored via the increase in light scattering at 360 nm at 37 °C as a function of time. All reactions were performed in 50 mM HEPES, pH 8, 200 mM KCl, and 2mM DTT buffer. Assays were acquired on a Synergy H1 microplate reader (BioTek) in an area scan mode with a 3x3 matrix for each well in clear, flat-bottom, 96-well plates (Nunc) sealed with optical adhesive film (Applied Biosystems). Signal was recorded every 80 seconds for 6-8 hours, with orbital continuous shaking (282 rpm) between measurements.

Insulin aggregation prevention assay

Insulin (Sigma-Aldrich) was dissolved in 50 mM HEPES pH 8.0, 200 mM KCl, 2.5% Acetic acid, and was dialysed in 50mM HEPES pH 8.0, 200mM KCl overnight. The dialysed Insulin (40 μM) was incubated in the presence or absence of JDPs (40 μM) for 10 minutes at 37 °C and the aggregation was induced by adding 3mM DTT and monitored by measuring light scattering at 360 nm as a function of time. All the JDPs were buffer exchanged to the reaction buffer 50 mM HEPES, pH 8, 200 mM KCl, and 2mM DTT buffer. The aggregation assay was conducted at 37 °C with orbital continuous shaking (282 rpm) on a BioTek Synergy H1 microplate reader in clear, flat-bottom, 96-well plates sealed with optical adhesive film (Applied Biosystems).

NMR experiments and analysis

All NMR experiments were carried out at 25 °C on 23.5 T (1,000 MHz) Bruker spectrometers equipped with triple-resonance single (z) or triple (x, y, z) gradient cryoprobes and Automatic Tuning and Matching. All NMR experiments were performed in 50 mM HEPES pH 7.0, 50 mM KCl, 10% D_2O. An ATP regeneration system (50 units of creatine kinase, 20 mM phosphocreatine, 5 mM ATP, and 10 mM $MgCl_2$) was added to all the experiments. The experiments were processed by NMRPipe and analyzed via NMRFAM-Sparky.

All the binding experiments were performed by acquiring a ^{1}H-^{15}N TROSY-HSQC spectrum. For all the interactions that were measured, two spectra were recorded, one for the relevant U-^{15}N J-domain alone (200μM) and another with the relevant unlabeled ATPase-deficient (T204A/ T205A) mutant of Hsp70 (50μM) in a ratio of 1:0.25.

Binding was determined by calculating the intensity ratios (I/I_0) from the peak height or peak volume that was fitted using the Gaussian fit in NMRFAM-Sparky software. Regions with signal loss greater than one standard deviation from the average intensity ratio were determined to be sites of binding.

NMR Assignment experiments

Backbone ^{1}H, ^{15}N and ^{13}C resonance assignments were carried out on a sample of 2 mM DNAJB5 in 50 mM HEPES pH 7.0, 50 mM KCl, 10% D_2O buffer. Assignments were obtained by recording a suite of 3D HNCACB, CBCA(CO)NH, HN(CA)CO, and HNCO experiments on a 600 MHz Bruker spectrometer. Unambiguous assignments of 99%, non-proline residues were achieved.

Assignments for J-domains of DNAJA1(10) (BMRB Entry 19163), DNAJA2 (1) (BMRB Entry 50169), DNAJB1 (1) (BMRB Entry 50169), DNAJB2(11) (BMRB Entry 17825), and DNAJB6 (4) (BMRB Entry 51996) were obtained from the corresponding BMRB entries.

Disaggregation of α-synuclein fibers

α-synuclein was purified as previously described (12). Next, α-synuclein fibrils were prepared by incubating 200 μM filter-sterilized α-syn monomers for one week in 25 mM $NaPO_4$ (pH 7.3), 100 mM NaCl, and 0.05% NaN_3 at 37 °C with shaking at 900 rpm (13). Aggregation into amyloid fibrils was confirmed by EM imaging and by ThT fluorescence. The disaggregation of the fibrils was measured by following the change in ThT fluorescence over 10 hours. Measurements were performed using a Synergy H1 microplate reader (BioTek), excitation: 440 nm, emission: 480 nm, 30 °C. The fibrils (2 μM) were pre-incubated for 30 minutes with 4 μM Hsp70, 2 μM DNAJB1, 0.2 μM HSP110, and 30 μM ThT at 30 °C in 50 mM HEPES-KOH pH 7.5), 50 mM KCl, 5 mM $MgCl_2$, and 2 mM DTT before measurement.

Cell culture and heat stress experiments

HEK293 cells were originally obtained from ATCC grown in DMEM (Gibco) and used as model system for cell biology studies, including western blot, biochemistry, and immunoprecipitation. Cells were routinely tested for mycoplasma contamination. Media were supplemented with 10% fetal bovine serum (Gibco), 1% Penicillin/streptomycin and L-glutamine (2 mmol/l) (Biological Industries). All cells were grown in a 37 °C with 5 % CO2 humidified incubator.

For heat stress experiments, cells were plated at 1.5 × 10^6 per 25 cm^2 flask (Greiner Bio-One Inc). Twenty-four hours later, cells were stressed for 1 h in a 42 °C water bath and returned to a 37 °C incubator for the indicated recovery time (20 h). Cell death was assessed by trypan blue staining and counted using the Countess II™ automated cell counter (Thermo Fisher Scientific).

NanoBiT Assay

HEK293T cells were maintained in Dulbecco's Modified Eagle Medium (DMEM; Gibco) supplemented with 10% fetal bovine serum (FBS; Gibco), 1% L-glutamine, 1% sodium pyruvate, and 1% penicillin-streptomycin. Cells were cultured at 37 °C in a humidified atmosphere containing 5% CO_2. For NanoBiT experiments, cells were seeded in 48-well plates at a density of 4 × 10^4 cells per well. After 24 h, cells were co-transfected in triplicate with 1.2 μg total plasmid DNA (0.6 μg of each construct) using Lipofectamine 3000 (Invitrogen), according to the manufacturer's instructions. For each triplicate transfection, 2.25 μl Lipofectamine 3000 reagent was diluted in 150 μl Opti-MEM, while 1.2 μg total DNA and 1.5 μl P3000 reagent were diluted in 146.5 μl Opti-MEM. The mixtures were combined and incubated for 30 min at room temperature before 100 μL of the transfection mixture was added to each well. Five hours post-transfection, the medium was replaced with fresh growth medium.

At 48 h post-transfection, cells were washed once with PBS and lysed in 50 μl NanoLuc lysis buffer (Promega) for 30 min at room temperature. Lysates were then transferred to white 96-well plates (Nunc). Luminescence was measured following the addition of 50 μl Nano-Glo substrate (Promega), diluted 1:50 in NanoLuc lysis buffer, using a Synergy H1 microplate reader (BioTek).

Data represent the mean ± SD of three independent experiments, each performed in technical triplicate.

Immuno-precipitation

HEK293 cells (25 cm^2 flask, ~60% confluency) were transiently transfected with either a V5-tagged protein of interest or a GFP control vector using the JetPEI DNA transfection reagent (Sartorius), following the manufacturer's instructions, and grown for 24 h. Cells were then heat stressed for 1 h in a 42 °C water bath and returned to a 37 °C incubator for the indicated recovery time (20 h).

Cells were washed twice with PBS and lysed in 50 mM HEPES, pH 7.5, 5% glycerol, 150 mM KCl, 10 mM MgCl2, 0.1% Triton-X, 2 mM ATP, and 1:400 protease-inhibitor mixture (Calbiochem) for 10 min on ice. The lysates were cleared by centrifugation for 10 min at 13,000g at 4 °C to remove cell debris. Protein concentration was assessed using BC Assay Protein Quantitation Kit (Interchim). 100 μg of cell lysate was then added to 10 μL of fresh Protein G-conjugated magnetic beads (GenScript), along with 1 μg of V5 antibody. The mixture was incubated overnight at 4°C with slow rotation, followed by three 1 ml washes with chilled TBS buffer. The target conjugates were then eluted from beads with 100 mM Tris-HCl, pH 8, 8 M urea, and 50 mM DTT for 30 min at 37 °C, and by adding protein loading dye (with reducing agents) and heating for 10 min at 95 °C, followed by denaturing immunoblotting.

Immunoblotting

Cells were washed twice with PBS and lysed in STET buffer (50 mM Tris-HCl, pH 87.5, 150 mM NaCl, 2 mM EDTA, 1% Triton-X, and 1:400 protease-inhibitor mixture (Calbiochem)) unless written otherwise. Protein concentration was assessed using BC Assay Protein Quantitation Kit (Interchim). 5-10 μg of total protein was separated by SDS–PAGE on 4-20% gradient ExpressPlus PAGE M42015 (GenScript) and transferred onto nitrocellulose membranes using iBlot™3 Gel Transfer Device (Thermo Fisher Scientific). The membranes were blocked in 5% skim milk prepared in TBS-0.1% Tween and incubated in primary antibodies overnight at 4°C followed by washing and incubation with secondary antibody. Blots were developed using SuperSignal™ West Pico PLUS Chemiluminescent Substrate (Thermo Fisher Scientific) and the ChemiDoc XRS+ Imaging System (Bio-Rad).

Antibodies

Primary antibodies used were as follows: mouse anti-V5 (46-0705, Invitrogen, LOT 3131596; 1:1000 for WB and 1:100 for IP), rabbit anti-DNAJA1 (ab192904, Abcam LOT 1147608-1; 1:1000 for WB), mouse anti-DNAJB1 (ab223607, Abcam; 1:1000 for WB), mouse anti-β-actin (ab170325, Abcam, LOT GR3242604-12; 1:5000 for WB), mouse anti-HSPA6/HSP76 (sc-374589, Santa Cruz Biotechnology, LOT J0824; 1:1000 for WB), mouse anti-HSPA6/HSP76 (ADI-SPA-754-D, Enzo, LOT 05012106; 1:1000 for WB), mouse anti-HSPA8 (sc-7298, Santa Cruz Biotechnology, LOT J2725; 1:1000 for WB), and rabbit anti-vinculin (13901, Cell Signaling Technology, LOT 10; 1:1000 for WB).

Statistical analysis

All quantified experiments were performed at least in triplicate. Data shown as mean ± s.d. and error bars indicating s.d. One-way ANOVA test was conducted to determine the significant differences between the two groups. All analyses were considered statistically significant at $p<0.05$.

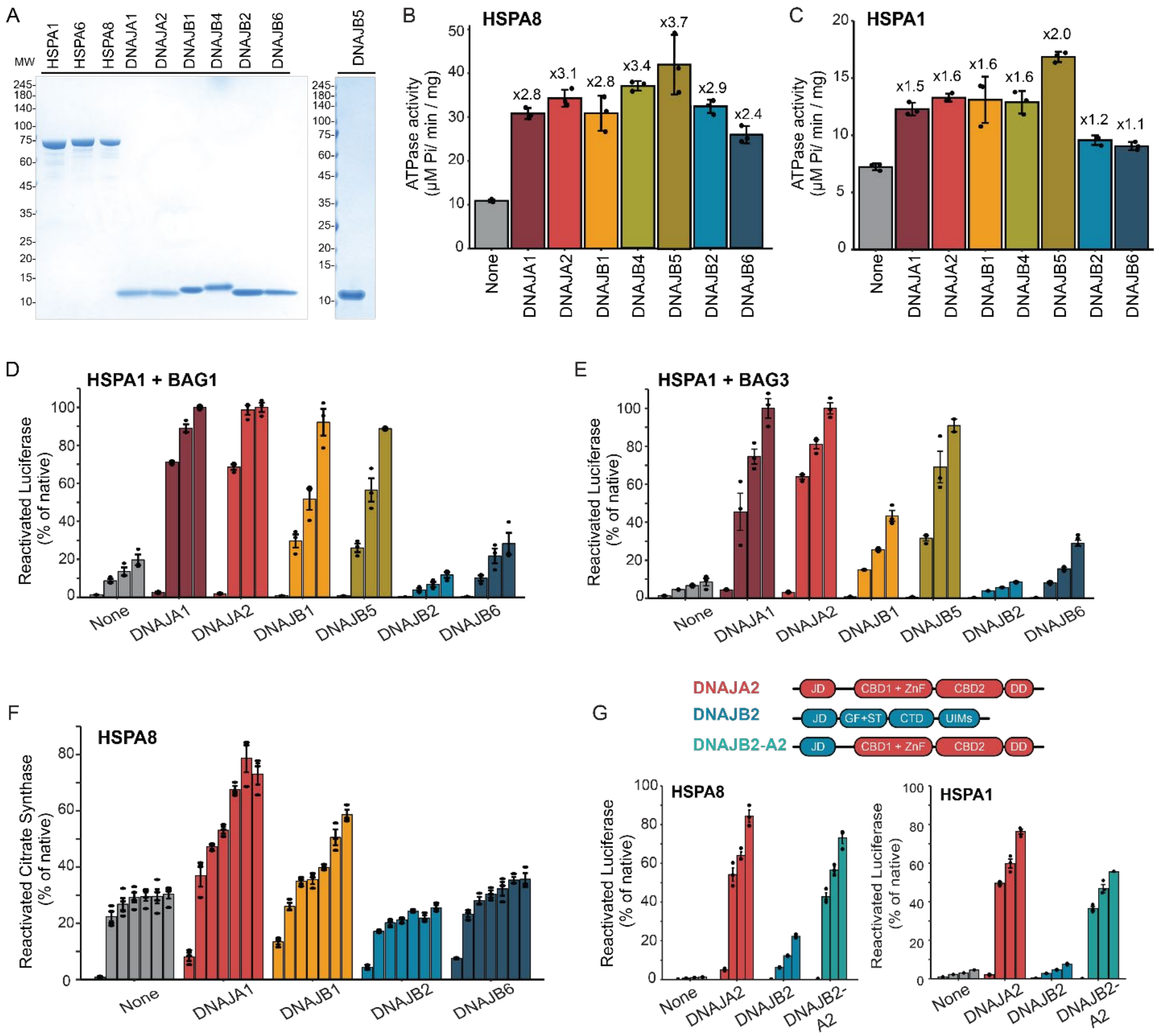

Figure S1. **Differential activity of different JDP classes with HSPA8 and HSPA1 chaperones.**

(A) SDS-PAGE analysis of purified Hsp70 chaperones and JDP J-domains used in this study. **(B-C)** Steady-state ATPase activity of HSPA8 (B) or HSPA1 (C) alone (grey) or with DNAJA1 (maroon), DNAJA2 (red), DNAJB1 (yellow), DNAJB4 (green), DNAJB5 (olive), DNAJB2 (blue), and DNAJB6 (dark blue) J-domains. All JDPs can activate HSPA8, enhancing its ATPase activity. Each bar represents the mean ± S.D. of three experiments. **(D-E)** Refolding activity of firefly luciferase (FFL) mediated by HSPA1 and either BAG1 (D) or BAG3 (E), in the absence (grey) or presence of DNAJA1 (maroon), DNAJA2 (red), DNAJB1 (yellow), and DNAJB5 (olive). FFL activity was measured at 0, 30, 60, and 120 minutes. Data points represent the mean ± S.D. of 3 independent measurements. **(F)** Refolding activity of citrate synthase mediated by HSPA8, Hsp110, and the indicated JDPs. DNAJA1 (maroon), DNAJA2 (red), DNAJB1 (yellow), DNAJB5 (olive), DNAJB2 (blue), or DNAJB6 (dark blue). Citrate synthase activity was measured at 0, 10,20, 30, 60, 90 and 120 minutes. Data points represent the mean ± S.D. of 3 independent measurements. (**G)** Domain organization of the WT DNAJA2 (red), WT DNAJB2 (blue), and the chimeric DNAJB2-A2 (turquoise), in which the DNAJB2 J-domain was fused to the client-binding domain of DNAJA2. Refolding assays of FFL by HSPA1 and HSPA8 were performed in the absence (grey) or presence of the indicated JDPs. FFL activity was measured at 0, 30, 60, and 120 minutes. Data points represent the mean ± S.D. of 3 independent measurements.

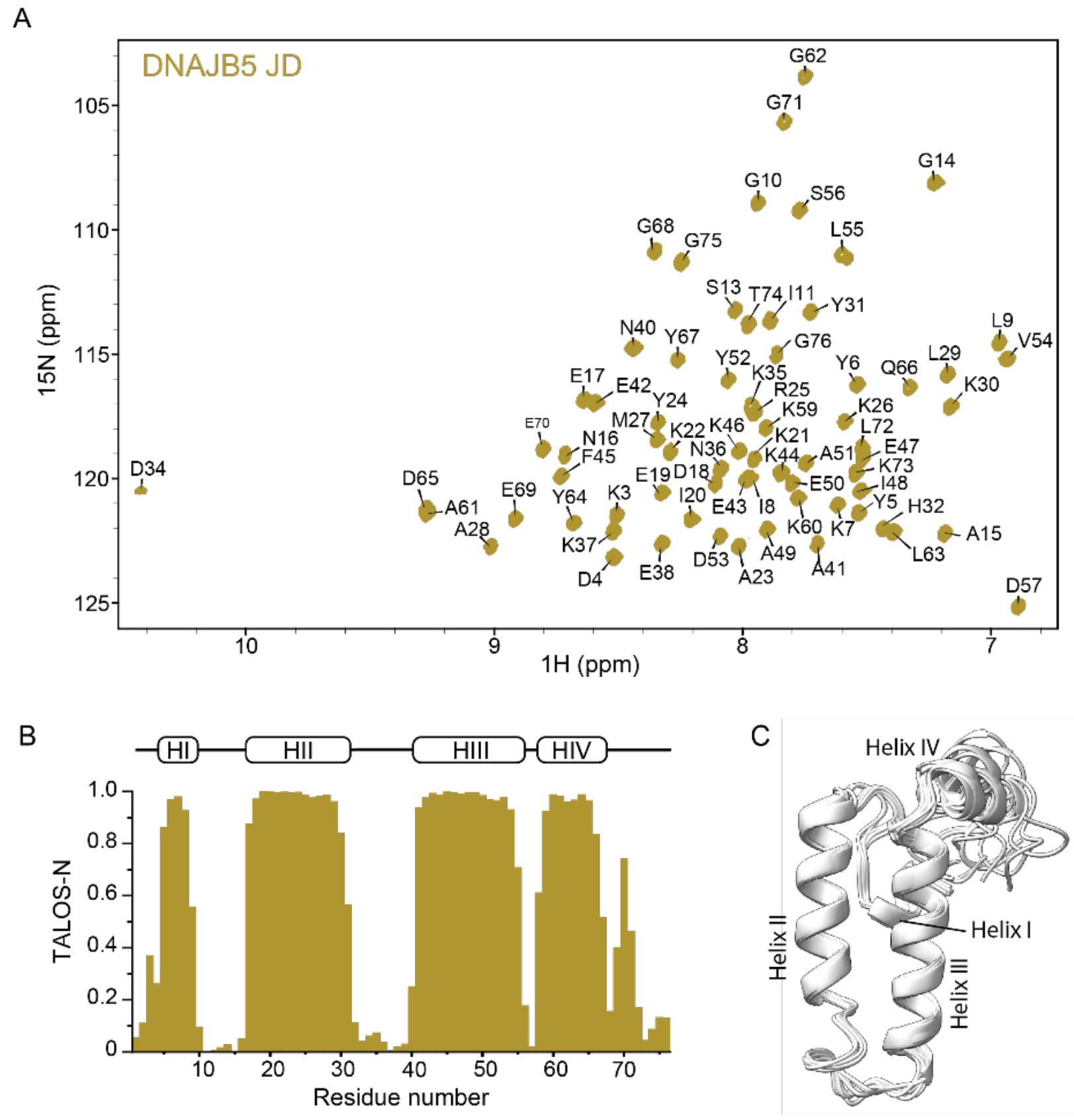

Figure S2. DNAJB5 J-domain construct. (A) ^{1}H-^{15}N HSQC spectra of ^{15}N-labeled DNAJB5 J-domain with residue assignments indicated. **(B)** TALOS-N secondary structure derived from ^{13}Cα, ^{13}Cβ, ^{13}C′, ^{15}N, and ^{1}HN chemical shifts showing the four characteristic α-helices of DNAJB5 J-domain. **(C)** Cartoon representation of the 10 lowest-energy solution NMR structures calculated by CS-Rosetta.

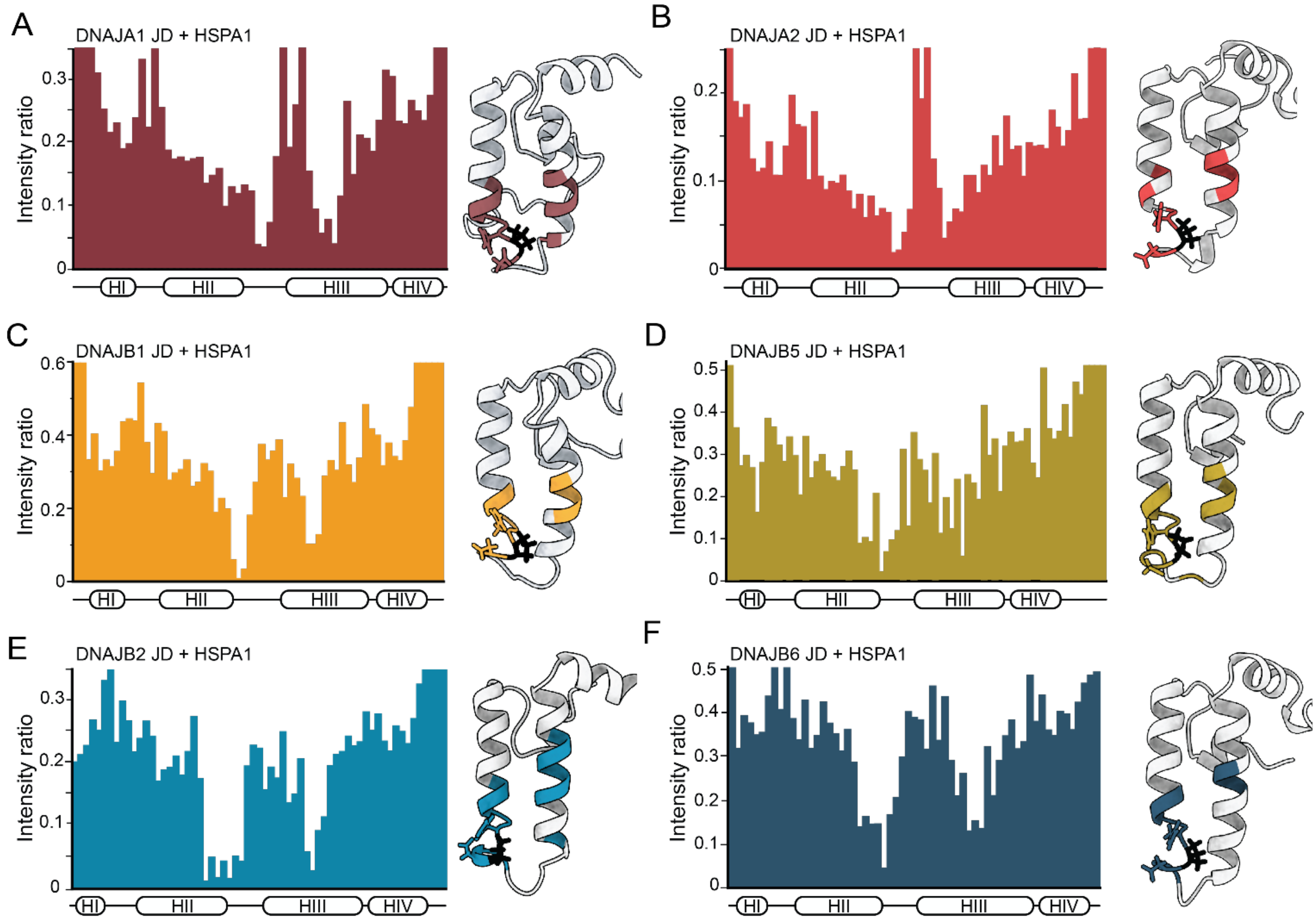

Figure S3. Structural characterization of HSPA1 and J-domains interactions. (A-F) Per-residue intensity ratios (I/I_0) for ^{15}N-labeled J-domains in the presence of HSPA1, where I and I_0 represent the HSQC peak intensities of the HSPA1-bound and free J-domains, respectively. Data are shown for DNAJA1 JD **(A)**, DNAJA2 JD **(B)**, DNAJB1 JD **(C)**, DNAJB5 JD **(D)**, DNAJB2 JD **(E)**, and DNAJB6 JD **(F)**, measured at a 1:0.25 J-domain:HSPA1 ratio. The positions of the four α-helices characteristic of J-domains are indicated below each plot. Substantial decreases in peak intensity are observed for residues located in helices **II** and **III** (HII and HIII) and in the flexible loop containing the conserved HPD motif, identifying the primary interaction interface with HSPA1. These interaction interfaces are highlighted on the corresponding J-domain structures: DNAJA1 JD (10), DNAJA2 JD (1), DNAJB1 JD (14), DNAJB5 (this study), DNAJB2 JD (11), and DNAJB6 JD (4).

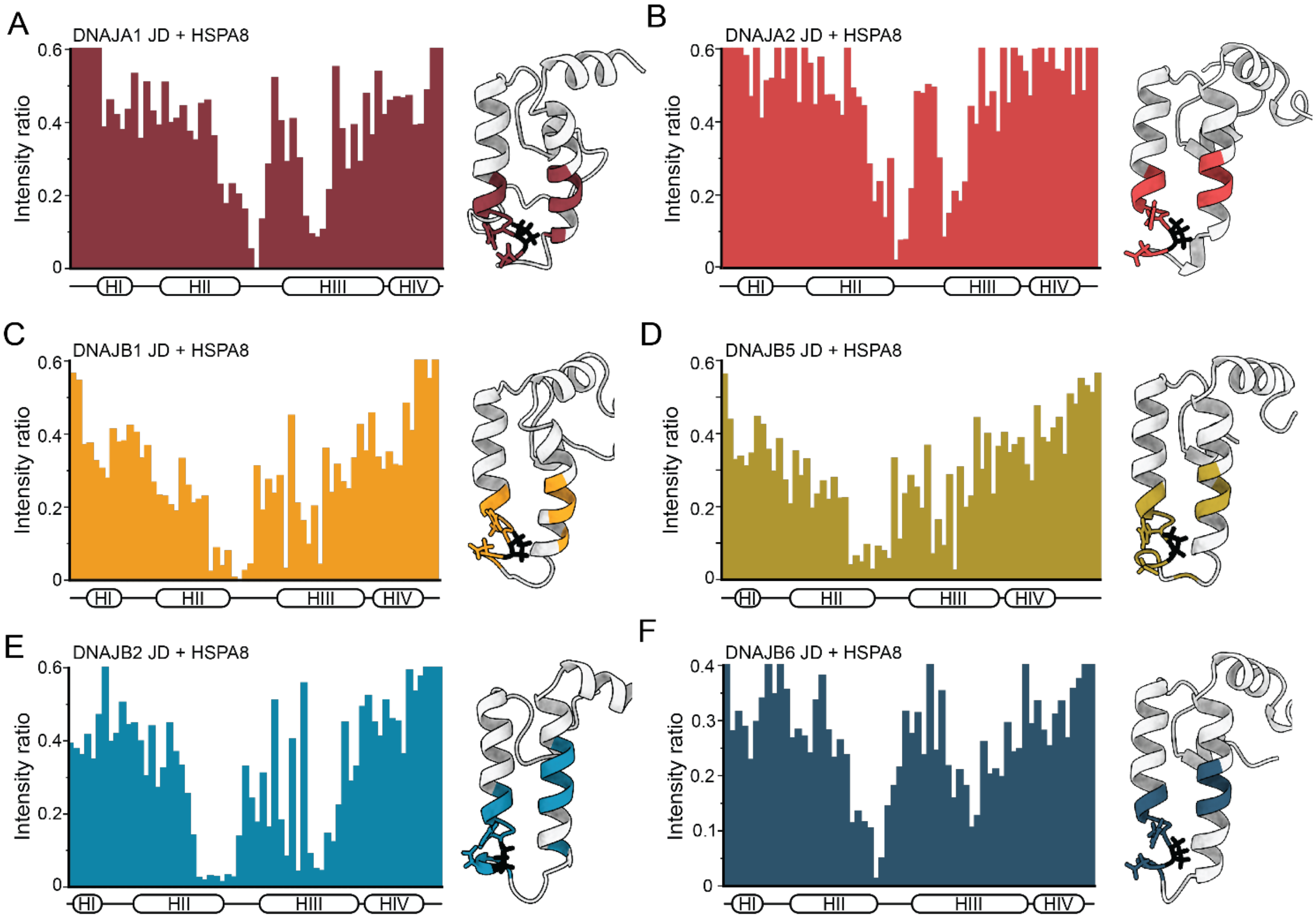

Figure S4. Structural characterization of HSPA8 and J-domains interactions. (A-F) Per-residue intensity ratios (I/I_0) for ^{15}N-labeled J-domains in the presence of HSPA8, where I and I_0 represent the HSQC peak intensities of the HSPA8-bound and free J-domains, respectively. Data are shown for DNAJA1 JD **(A)**, DNAJA2 JD **(B)**, DNAJB1 JD **(C)**, DNAJB5 JD **(D)**, DNAJB2 JD **(E)**, and DNAJB6 JD **(F)**, measured at a 1:0.25 J-domain:HSPA8 ratio. The positions of the four α-helices characteristic of J-domains are indicated below each plot. Substantial decreases in peak intensity are observed for residues located in helices II and III (HII and HIII) and in the flexible loop containing the conserved HPD motif, identifying the primary interaction interface with HSPA8. These interaction interfaces are highlighted on the corresponding J-domain structures: DNAJA1 JD (10), DNAJA2 JD (1), DNAJB1 JD (14), DNAJB5 (this study), DNAJB2 JD (11), and DNAJB6 JD (4).

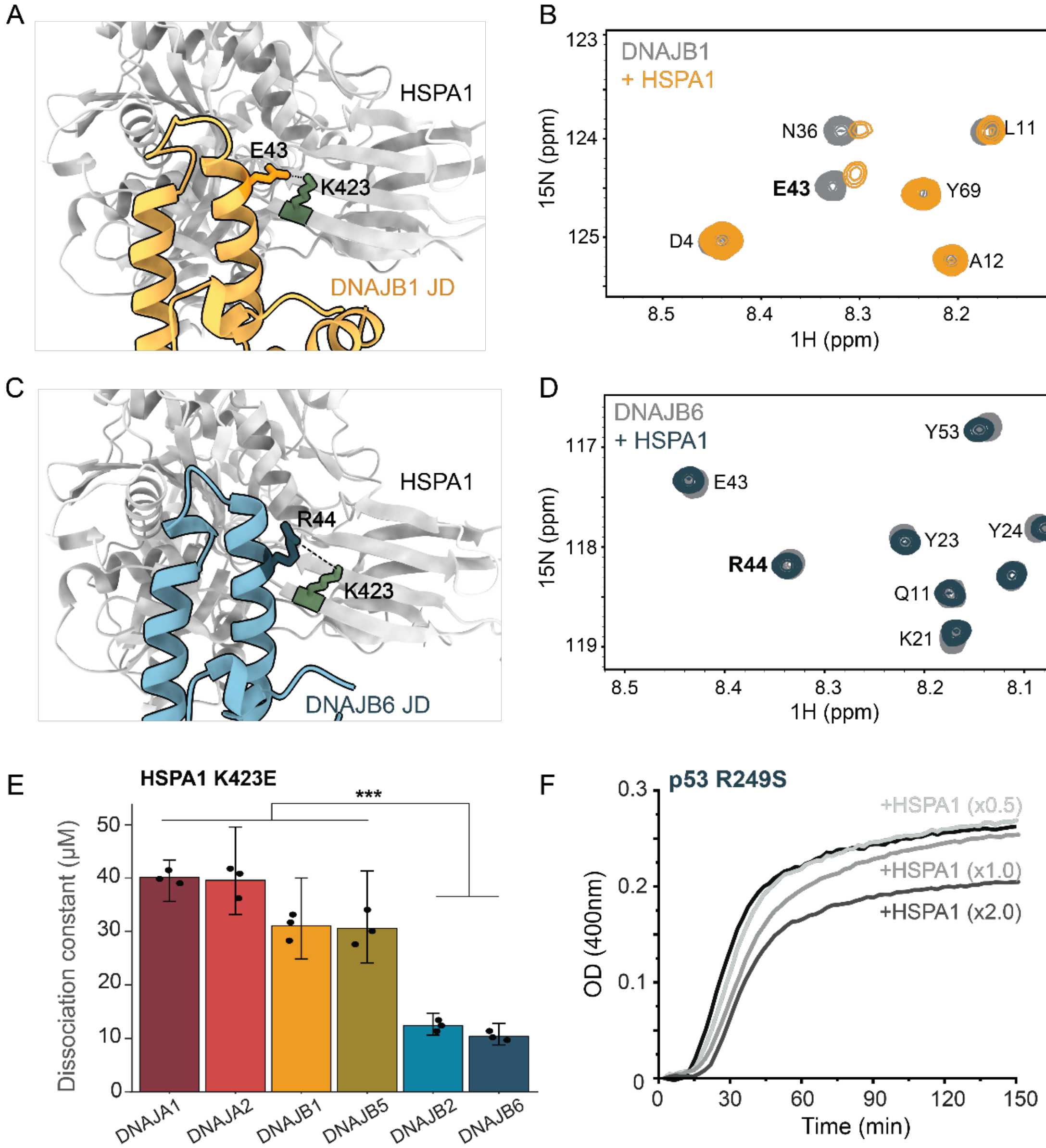

Figure S5. Charge complementarity at the J-domain–HSPA1 interface governs binding specificity. (A) AlphaFold structural model of the DNAJB1 J-domain (yellow) bound to HSPA1 (grey), illustrating the electrostatic interaction between DNAJB1 residue E43 and HSPA1 residue K423 located in the SBDβ. **(B)** ^{1}H-^{15}N HSQC spectra of DNAJB1 JD alone (grey) or in the presence of HSPA1 (yellow). Significant reduction in intensity and chemical shift perturbation is observed for residue E43, consistent with productive binding. **(C)** AlphaFold structural model of HSPA1 bound to the DNAJB6 J-domain (dark blue), highlighting the electrostatic repulsion between the similarly charged residues R44 (DNAJB6) and K423 (HSPA1). **(D)** ^{1}H-^{15}N HSQC spectra of free (grey) and HSPA1-bound DNAJB6 JD (dark blue), showing that residue R44 of DNAJB6 shows minimal perturbation upon HSPA1 addition. **(E)** Fluorescence polarization measurements of DNAJA1 (maroon), DNAJA2 (red), DNAJB1 (yellow), DNAJB5 (olive), DNAJB2 (blue), and DNAJB6 (dark blue) J-domains binding to HSPA1 K423E mutant. Data points represent the mean ± 95% confidence intervals calculated from log-transformed K_D values for 3 independent measurements. The HSPA1 mutant displayed a reverse binding pattern to that of the WT HSPA1 protein with higher affinities measured for Class B' JDPs relatively to those of the canonical JDPs. (**F**) Aggregation kinetics of mutant p53 R249S monitored at 400 nm in the absence of HSPA1 (black) or in the presence of increasing concentrations of HSPA1 (light to dark grey). This experiment serves as a control for Fig. 3C and demonstrates the dose-dependent aggregation prevention activity of HSPA1.

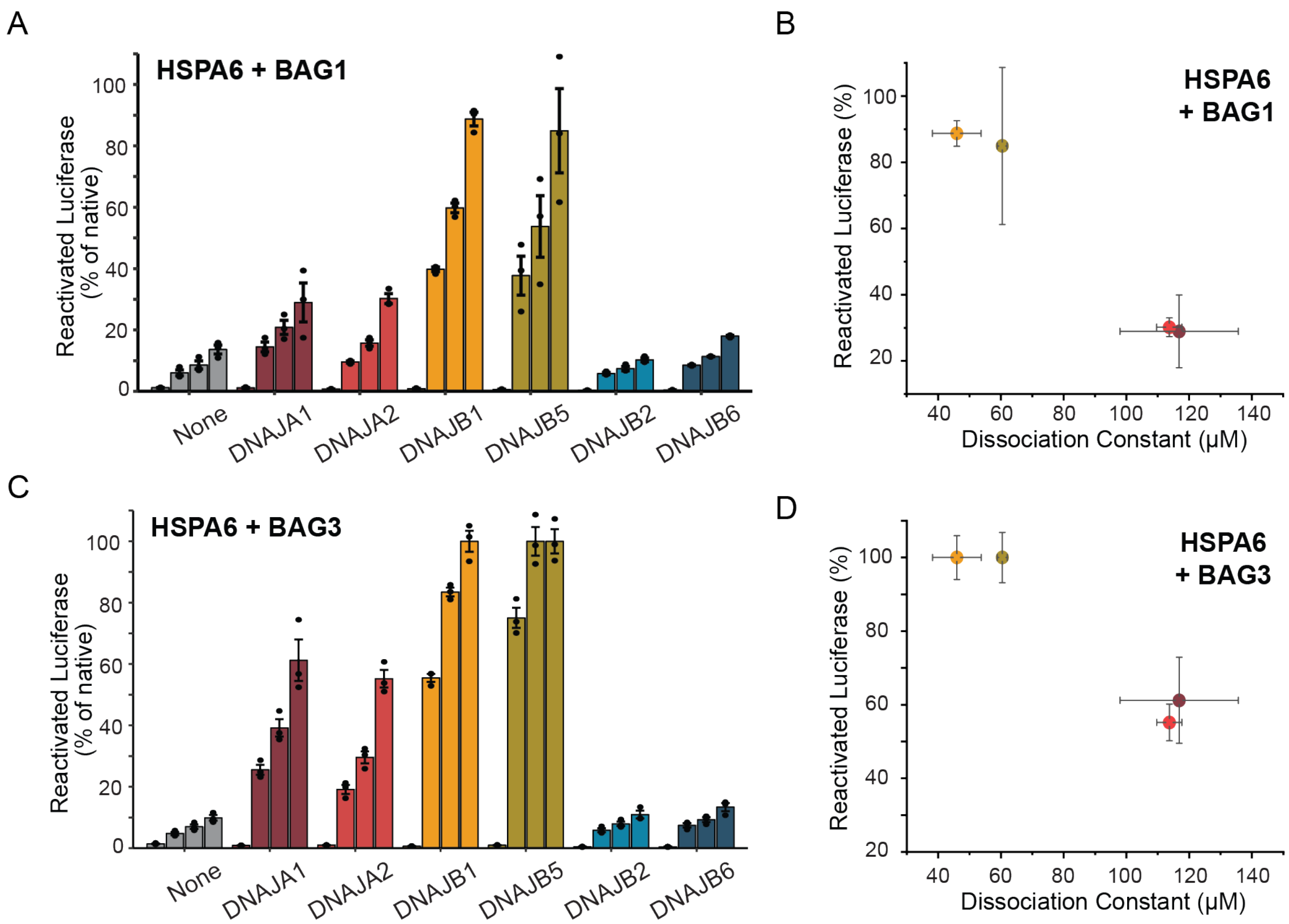

Figure S6. HSPA6 refolding activity as a function of different NEFs. (A, C) Refolding activity of FFL mediated by HSPA6 and either BAG1 **(A)** or BAG3 **(C)**, in the absence (grey) or presence of DNAJA1 (maroon), DNAJA2 (red), DNAJB1 (yellow), and DNAJB5 (olive). FFL activity was measured at 0, 30, 60, and 120 minutes. Data points represent the mean ± S.D. of 3 independent measurements. **(B, D)** Correlation between the dissociation constants (K_D) of the J-domains binding to HSPA6 working with either BAG1 **(B)** or BAG3 **(D)**, and the ability of the corresponding full-length JDPs to support HSAP6-dependent FFL refolding activity

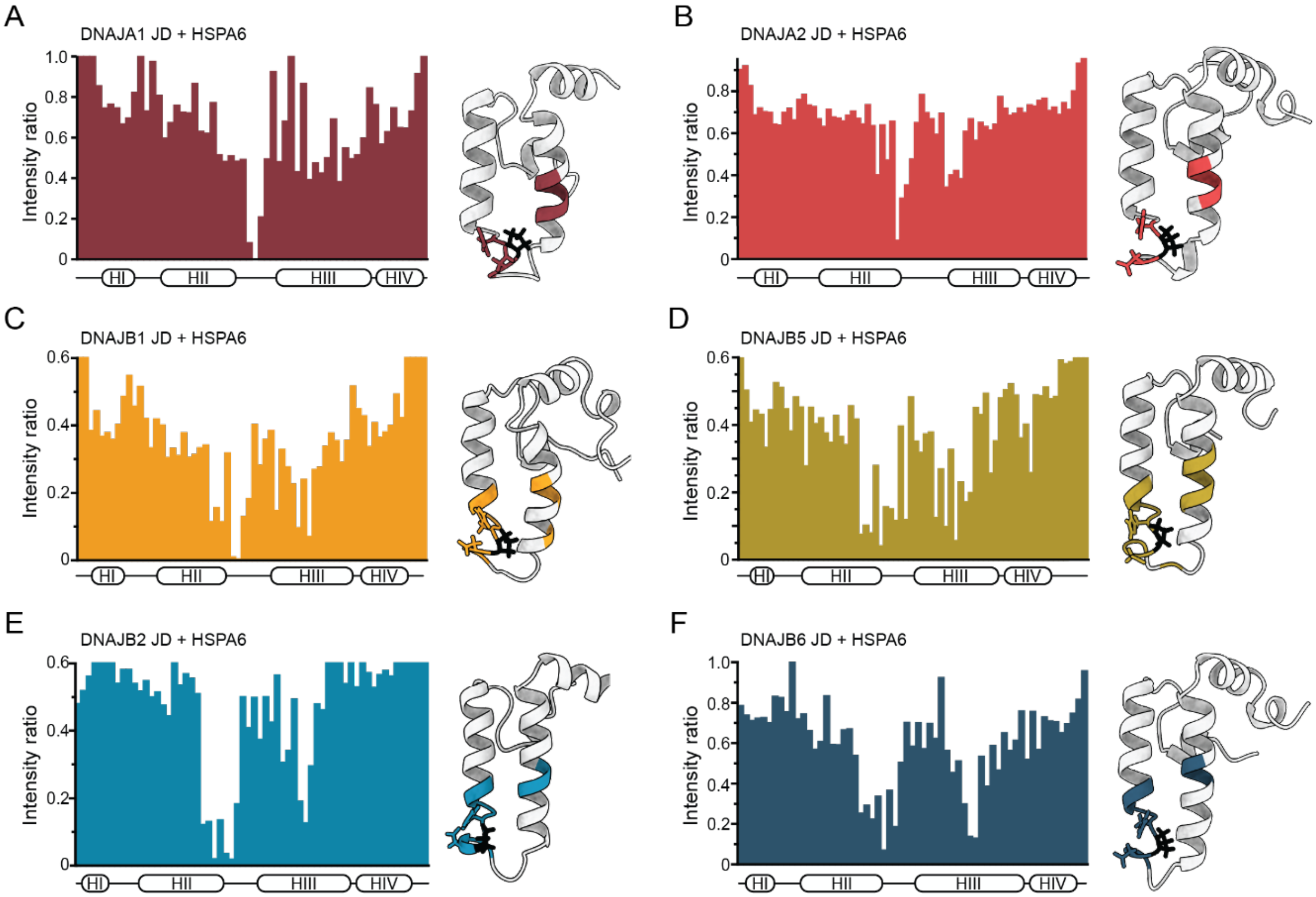

Figure S7. Structural characterization of HSPA6 and J-domains interactions. (A-F) Per-residue intensity ratios (I/I_0) of ^{15}N-labeled J-domains of DNAJA1 **(A)**, DNAJA2 **(B)**, DNAJB1 **(C)**, DNAJB5 **(D)**, DNAJB2 **(E)**, and DNAJB6 **(F)**, measured alone (I_0) or in the presence of HSPA6 (I). Secondary structure elements of each J-domain indicated at the bottom of each plot. Strong reductions in signal intensity are observed for residues located within helices II and III and the loop containing the conserved HPD motif, highlighting these regions as interaction sites. Clear exceptions are DNAJA1 and DNAJA2 J-domains that show significantly weaker interaction only at helix **III** and the HPD. The identified interaction sites are mapped onto J-domain structures - DNAJA1 JD (10), DNAJA2 JD (1), DNAJB1 JD (14), DNAJB5 (this study), DNAJB2 JD (11), and DNAJB6 JD (4).

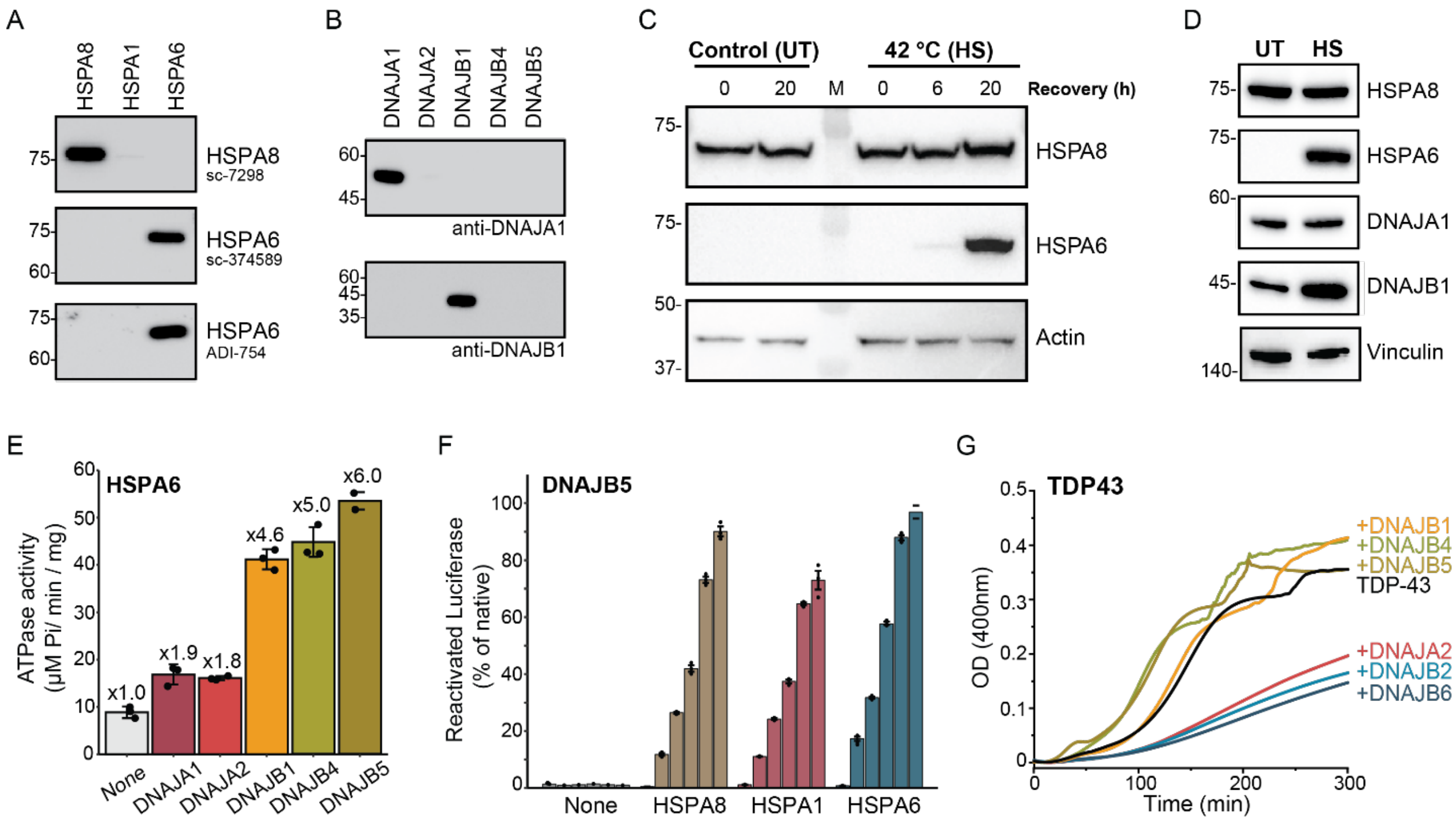

Figure S8. HSPA6 functions are determined by its affinity toward the J-domains. (A-B) Validation of the specificity of commercially available antibodies against different Hsp70 and JDP paralogs using purified recombinant proteins. The antibodies used throughout this study showed specific recognition of HSPA8, HSPA6, DNAJA1, or DNAJB1, with no cross-reactivity between paralogs. **(C)** HSPA6 expression is strongly induced following cellular stress. Western blot analysis of cell lysates collected at 0, 6 or 20 h after heat shock (42°C) shows that HSPA6 levels are undetectable under basal conditions but become highly upregulated following stress, whereas HSPA8 levels remain largely unchanged. **(D)** Cellular response of chaperone expression to a 1 h heat shock at 42 °C. Western blot analysis using the paralog-specific antibodies (validated in panels A–B) demonstrates that HSPA8 and DNAJA1 protein levels remain relatively constant following heat shock, whereas HSPA6 and DNAJB1 are strongly induced. **(E)** Steady-state ATPase activity of HSPA6 alone (grey) or with DNAJA1 (maroon), DNAJA2 (red), DNAJB1 (yellow), DNAJB4 (green olive), or DNAJB5 (olive) J-domains. Each bar represents the mean ± S.D. of three experiments. **(F)** Refolding activity of firefly luciferase (FFL) mediated by DNAJB5, Hsp110, and the indicated Hsp70 chaperones - HSPA8 (brown), HSPA1 (pink), or HSPA6 (blue). FFL activity was measured at 0, 10, 20, 30, 50, and 60 minutes. Data points represent the mean ± S.D. of 3 independent measurements. **(G)** Aggregation of TDP-43 (6 µM) monitored in the absence (black) or presence of 12 µM DNAJA2 (red), DNAJB1 (yellow), DNAJB4 (green), DNAJB5 (olive), DNAJB2 (blue), or DNAJB6 mutant (dark blue). Class B JDPs (DNAJB1, DNAJB4, DNAJB5) do not prevent TDP-43 aggregation, whereas Class A and Class B′ JDPs are functional as TDP-43 holdases. Data points represent the mean ± S.D. of 3 independent measurements.

Table S1. Affinities measured for the various JDP-Hsp70 pairs.

	JDPs	HSPA8 (μM)	HSPA1 (μM)	HSPA6 (μM)
Class A	DNAJA1	14.5±3.0 (95%CI: 8.8−23.8)	24.8±6.1 (95% CI: 18.9-30.5)	112.7±13.0 (95% CI: 97.2-129.3)
	DNAJA2	12.5±0.4 (95%CI: 11.6−13.4)	20.5±4.0 μM (95% CI: 15.7–26.0)	113.7±14.0 (95% CI: 98.76-128.7)
Class B	DNAJB1	15.8±2.9 (95%CI: 12.3−19.3)	20.0±5.6 (95% CI: 13.7-26.9)	45.9±7.8 (95% CI: 36.8–55.9)
	DNAJB5	18.8±1.0 (95%CI: 16.3−21.5)	32.3±3.8 (95% CI: 27.8-37.2)	50.4±4.0 (95% CI: 46.5–54.6)
Class B'	DNAJB2	27.1±4.4 (95%CI: 21.8−33.0)	65.6±8.5 (95% CI: 55.2–76.7)	114.0±3.9 (95% CI: 109.3–118.9)
	DNAJB6	24.4±1.1 (95%CI: 21.3−27.9)	72.9±7.9 (95% CI, 63.5–82.8)	126.7±19.4 (95% CI: 106.8–138.3)

Dissociation constants were obtained by fitting each independent fluorescence polarization titration separately. Standard deviations are provided for comparison with previous studies, while the corresponding 95% confidence intervals were calculated from log-transformed K_D values and represent the statistical uncertainty of the estimated K_D of 3-5 independent experiments.

Table S2. Affinities measured for the various mutant JDP-Hsp70 pairs.

JDPs:Hsp70	K_D (μM)
DNAJB2 K44E – HSPA1	36.0±5.3 (95% CI: 30.5-41.9)
DNAJB2 K44L – HSPA1	68.2±20.4 (95% CI: 37.0-100.1)
DNAJB6 R44E – HSPA1	46.2±1.1 (95% CI: 44.7-47.7)
DNAJB6 R44L – HSPA1	96.2±7.9 (95% CI: 73.2-125.7)

JDPs:Hsp70	K_D (μM)
DNAJA2 L31Q – HSPA6	128.3±6.5 (95% CI: 112.0–146.7)
DNAJA2 P41K – HSPA6	110.7±37.4 (95% CI: 68.7–158.9)
DNAJA2 G44E – HSPA6	143.7±32.5 (95% CI: 90.8–213.6)
DNAJA2-B1 loop – HSPA6	96.5±9.1 (95% CI: 85.4–108.2)
DNAJA2-B1-HII-loop	50.8±4.4 (95% CI: 45.4–56.4)

DNAJA1-HSPA1 K423E	40.1±1.2 (95% CI: 36.4-44.2)
DNAJA2-HSPA1 K423E	39.6±3.0 (95% CI: 31.7-49.2)
DNAJB1-HSPA1 K423E	31.0±2.5 (95% CI: 24.6-39.1)
DNAJB5-HSPA1 K423E	30.4±3.3 (95% CI: 22.4-41.1)
DNAJB2-HSPA1 K423E	12.4±1.0 (95% CI: 9.8-15.6)
DNAJB6-HSPA1 K423E	10.4±0.9 (95% CI: 8.5-12.7)

Dissociation constants were obtained by fitting each independent fluorescence polarization titration separately. Standard deviations are provided for comparison with previous studies, while the corresponding 95% confidence intervals were calculated from log-transformed K_D values and represent the statistical uncertainty of the estimated K_D of 3-5 independent experiments.

Table S3. Cellular levels of JDPs and Hsp70 paralogs and the upper-limit estimated fraction bound under stress conditions.

	Cellular conc [nM]	
	**No Stress**	**Stress 3h (15)**
DNAJA1	2900	6090
DNAJA2	1100	1210
DNAJB1	800	63680
DNAJB5	9	10
DNAJB2	15	12
DNAJB6	770	1232
HSPA8	13000	22100
HSPA1A	3	10299
HSPA1B	6200	49600
HSPA6	0	30336

	Percentage Bound (%) *			
	**HSPA8**		**HSPA1**	**HSPA6**
	**No Stress**	**stress 3h**	**stress 3h**	**stress 3h**
DNAJA1	10%	15%	8%	4%
DNAJA2	4%	4%	2%	0.8%
DNAJB1	3%	75%	62%	51%
DNAJB5	0.03%	0.02%	0.12%	0.1%
DNAJB2	0.04%	0.02%	0.1%	0.1%
DNAJB6	2%	3%	1%	0.8%

* The upper-limit estimate assuming complete binding of the entire cellular pool of a given JDP to the corresponding Hsp70 paralog.

Intracellular concentrations of Hsp70s and JDPs under non-stress conditions in HEK cells were obtained from the open-source database OpenCell database (16, 17). Stress-induced concentrations were estimated by applying the fold changes reported by Hageman et al. following a 30-min heat shock at 45°C and 3 h recovery (18). For HSPA6, stress-induced concentrations were estimated based on its relative abundance compared to HSPA8 and scaled according to the estimated stress-induced concentration of HSPA8. Bound fractions were calculated using steady-state equilibrium binding equations based on the estimated intracellular concentrations and the experimentally determined K_D values (SI Appendix, Table S1).

SI References

1. O. Faust *et al.*, HSP40 proteins use class-specific regulation to drive HSP70 functional diversity. *Nature* **587**, 489-494 (2020).
2. W. Meng, E. M. Clerico, N. McArthur, L. M. Gierasch, Allosteric landscapes of eukaryotic cytoplasmic Hsp70s are shaped by evolutionary tuning of key interfaces. *Proc Natl Acad Sci U S A* **115**, 11970-11975 (2018).
3. G. Zoltsman *et al.*, A unique chaperoning mechanism in class A JDPs recognizes and stabilizes mutant p53. *Mol Cell* **84**, 1512-1526 e1519 (2024).
4. M. Abayev-Avraham, Y. Salzberg, D. Gliksberg, M. Oren-Suissa, R. Rosenzweig, DNAJB6 mutants display toxic gain of function through unregulated interaction with Hsp70 chaperones. *Nat Commun* **14**, 7066 (2023).
5. L. H. Arnold, S. Kunzelmann, M. R. Webb, I. A. Taylor, A continuous enzyme-coupled assay for triphosphohydrolase activity of HIV-1 restriction factor SAMHD1. *Antimicrob Agents Chemother* **59**, 186-192 (2015).
6. H. Rampelt *et al.*, Metazoan Hsp70 machines use Hsp110 to power protein disaggregation. *EMBO J* **31**, 4221-4235 (2012).
7. E. M. Quandt *et al.*, Fine-tuning citrate synthase flux potentiates and refines metabolic innovation in the Lenski evolution experiment. *Elife* **4** (2015).
8. P. Wang *et al.*, Reciprocal Regulation of the TOR Kinase and ABA Receptor Balances Plant Growth and Stress Response. *Mol Cell* **69**, 100-112 e106 (2018).
9. J. N. Rauch, J. E. Gestwicki, Binding of human nucleotide exchange factors to heat shock protein 70 (Hsp70) generates functionally distinct complexes in vitro. *J Biol Chem* **289**, 1402-1414 (2014).
10. J. L. Stark *et al.*, Structure and function of human DnaJ homologue subfamily a member 1 (DNAJA1) and its relationship to pancreatic cancer. *Biochemistry* **53**, 1360-1372 (2014).
11. X. C. Gao *et al.*, The C-terminal helices of heat shock protein 70 are essential for J-domain binding and ATPase activation. *J Biol Chem* **287**, 6044-6052 (2012).
12. M. P. Hinault *et al.*, Stable alpha-synuclein oligomers strongly inhibit chaperone activity of the Hsp70 system by weak interactions with J-domain co-chaperones. *J Biol Chem* **285**, 38173-38182 (2010).
13. A. S. Wentink *et al.*, Molecular dissection of amyloid disaggregation by human HSP70. *Nature* **587**, 483-488 (2020).
14. Y. Q. Qian, D. Patel, F. U. Hartl, D. J. McColl, Nuclear magnetic resonance solution structure of the human Hsp40 (HDJ-1) J-domain. *J Mol Biol* **260**, 224-235 (1996).
15. J. Hageman, H. H. Kampinga, Computational analysis of the human HSPH/HSPA/DNAJ family and cloning of a human HSPH/HSPA/DNAJ expression library. *Cell Stress Chaperones* **14**, 1-21 (2009).
16. J. R. Wisniewski, M. Y. Hein, J. Cox, M. Mann, A "proteomic ruler" for protein copy number and concentration estimation without spike-in standards. *Mol Cell Proteomics* **13**, 3497-3506 (2014).
17. N. H. Cho *et al.*, OpenCell: Endogenous tagging for the cartography of human cellular organization. *Science* **375**, eabi6983 (2022).
18. J. Hageman, M. A. van Waarde, A. Zylicz, D. Walerych, H. H. Kampinga, The diverse members of the mammalian HSP70 machine show distinct chaperone-like activities. *Biochem J* **435**, 127-142 (2011).
